# The ACPYPE web server for small-molecule MD topology generation

**DOI:** 10.1093/bioinformatics/btad350

**Published:** 2023-05-30

**Authors:** Luciano Kagami, Alan Wilter, Adrian Diaz, Wim Vranken

**Affiliations:** Interuniversity Institute of Bioinformatics in Brussels, VUB/ULB, Brussels 1050, Belgium; Phenopolis Ltd & Moorfields Eye Hospital, London, United Kingdom; Interuniversity Institute of Bioinformatics in Brussels, VUB/ULB, Brussels 1050, Belgium; Structural biology Brussels, Vrije Universiteit Brussel, Brussels 1050, Belgium; Interuniversity Institute of Bioinformatics in Brussels, VUB/ULB, Brussels 1050, Belgium; Structural biology Brussels, Vrije Universiteit Brussel, Brussels 1050, Belgium

## Abstract

**Motivation:**

The generation of parameter files for molecular dynamics (MD) simulations of small molecules that are suitable for force fields commonly applied to proteins and nucleic acids is often challenging. The ACPYPE software and website aid the generation of such parameter files.

**Results:**

ACPYPE uses OpenBabel and ANTECHAMBER to generate MD input files in Gromacs, AMBER, CHARMM, and CNS formats. It can now take a SMILES string as input, in addition to the original PDB or mol2 coordinate files, with GAFF2 support and GLYCAM force field conversion added. It can be installed locally via Anaconda, PyPI, and Docker distributions, while the web server at https://bio2byte.be/acpype/ was updated with an API, and provides visualization of results for uploaded molecules as well as a pre-generated set of 3738 drug molecules.

**Availability and implementation:**

The web application is freely available at https://www.bio2byte.be/acpype/ and the open-source code can be found at https://github.com/alanwilter/acpype.

## 1 Introduction

Molecular dynamics (MD) simulations provide information about the time-dependent evolution of molecular systems at the atomic level. In biology, MD simulations are widely used to study the conformations, dynamics, and interactions of proteins and nucleic acids and provide important information about the variability in these systems (Kagami *et al.* 2017). While most of the commonly used MM force fields such as CHARMM (Vanommeslaeghe *et al.* 2009), OPLS-AA (Kaminski *et al.* 2001), GROMACS (Van Der Spoel *et al.* 2005), and AMBER (Wang *et al.* 2004) provide complete parameters for proteins and nucleic acids, the correct parameterization of many ligands remains a challenge with these force fields, as they are not optimized for small molecules. To simplify the inclusion of ligands in biological molecular systems, ACPYPE was developed in 2012. ACPYPE is a Python wrapper script around the ANTECHAMBER software that aids the generation of small-molecule topologies and parameters for a variety of MD programs by providing input files in AMBER, CHARMM, GROMACS, and CNS formats (Phillips *et al.* 2005). It is widely used in the community, with currently almost 1500 published papers referring to ACPYPE. During its extensive use by the community, ACPYPE has continually been updated in pace with GROMACS and AmberMD releases and we are now announcing the updated ACPYPE version and server (https://bio2byte.be/acpype/). Notably, in 2019, the ability to convert AMBER GLYCAM06 force field to GROMACS was added to ACPYPE by Bernardi *et al.* (2019). ACPYPE now also supports input in SMILES format, has GAFF2 support, and is included in the GROMACS user guide (https://manual.gromacs.org/current/user-guide/index.html), as the tool to generate AMBER-based topologies. ACPYPE is now available for local install from GitHub, Anaconda, Pypi, and Docker Hub. It has in addition been embedded in MD simulation frameworks such as SimTK/mmtools (https://simtk.org/home/mmtools), MemGen (http://memgen.uni-goettingen.de/), LiGRO (Kagami *et al.* 2017), PolyParGen (http://polypargen.com/), Cosolvent Analysis Toolkit (Sabanés Zariquiey, de Souza and Bronowska 2019), BioExcel Building Blocks (https://mmb.irbbarcelona.org/biobb/), and Winmostar V10 (https://winmostar.com). The new server uses a token-based system, has API call functions, provides pre-generated files for common ligands, and supports direct access to all files as well as visualization of atom and coordinate information, including InChI information and links to online chemical resources (when available). In addition, the new functionality enables easier manual verification of the ACPYPE results, such as visualizing the coordinates to check, for example, for possible missing improper dihedrals in aromatic systems. We also refer to the GROMACS and AmberMD mailing lists when help in parametrization is required.

## 2 Materials and methods

### 2.1 Input

ACPYPE accepts 3 input atom coordinate formats, using the Openbabel package (https://openbabel.org/) to enable the upload of SYBYL MOL2 (https://www.certara.com/) and PDB (Protein Data Bank) formats (Shao *et al.* 2022), as well as text entry of SMILES (Simplified Molecular Input Line Entry System) (O’Boyle 2012). Entries of up to 200 atoms are accepted on the server.

### 2.2 Output

ACPYPE outputs 16 different file formats in a single run (Table 1), both from Amber and Charmm.

**Table 1.** ACPYPE output files.

Amber	Charmm	CNS	GROMACS	Coordinates	Other
INPCRD	INP	INP	ITP^a^	MOL2	MDP^b^
LIB	PRM	PAR	TOP^a^	PDB^c^	LOG^d^
PMRTOP	RTF	TOP	GRO		

a Amber and OPLS force field.

b GROMACS parameters files to run minimization and dynamics.

c Modified atom numbering.

d ACPYPE run logs.

### 2.3 RESTful API

The ACPYPE API uses the Django REST Framework 3.11 and provides programmatic access to submission of single coordinate file in PDB and MOL2 in file format (recommended) or a single SMILES in string format, which is less reliable as 3D coordinates for the atoms must be generated through OpenBabel (O’Boyle 2012). Using two endpoints (Table 2), the API returns the results in JSON (JavaScript Object Notation) format. The result can be retrieved using the alphanumeric hash ID provided by the submission process, so protecting the users' data security and confidentiality. Batch processes are possible by submission of a single job at a time.

**Table 2.** ACPYPE REST API endpoints.

Method	Endpoint	Description
POST	/acpype/api/	Sends the data for processing.
GET	/acpype/api/queue/	Gets the data after processing.^a^

a Using the alphanumeric hash ID provided during submission.

### 2.4 SMILES format recognition and ACPYPE performance

The ACPYPE functionality to directly use SMILES strings depends on converting this format to a three-dimensional model, which is performed by the embedded OpenBabel software. To verify how this new function handles the different SMILES, we ran ACPYPE for 4099 different drugs obtained from the DrugCentral server (Avram *et al.* 2021). For this, a TSV file was downloaded and used together with a Python script to convert SMILES to MOL2 format. The ability of ACPYPE to parameterize these MOL2 files was then tested, with all log files saved and analyzed.

### 2.5 Local installation availability

ACPYPE can also be installed locally from GitHub (https://github.com/alanwilter/acpype) as well as via Anaconda (https://anaconda.org/conda-forge/acpype), Pypi (https://pypi.org/project/acpype/), and Docker Hub (https://hub.docker.com/r/acpype/acpype). All ACPYPE functionality can be obtained using the local installation, including direct SMILES conversion. The source version also does not limit runtime or number of atoms.

## 3 Results

The updates on the features, local installation options, and server extensions of ACPYPE are aimed at making the software easier to use and more accessible to users. Particularly useful might be the ACPYPE SMILES functionality, which was able to handle 91.2% of a set of small drug molecules (totalling 3738 compounds—Table 3). Failures are due to limitations of the ANTECHAMBER program (0.4%), or due to checks included in the ACPYPE program such as failures in the recognition of intramolecular bonds (1%), detection of multiple molecules (2.9%), or maximum processing time of 3 h exceeded (4.4%). The complete table with results is reported in Supplementary Tables S1 and S2.

**Table 3.** ACPYPE API success rate for SMILES format submission.

Status	Number of compounds	%
Success	3738	91.2
Antechamber failed	18	0.4
Intramolecular bonds	42	1.0
More than one molecule detected	120	2.9
Time exceeded	181	4.4
Total	4099	100

**Figure 1.**
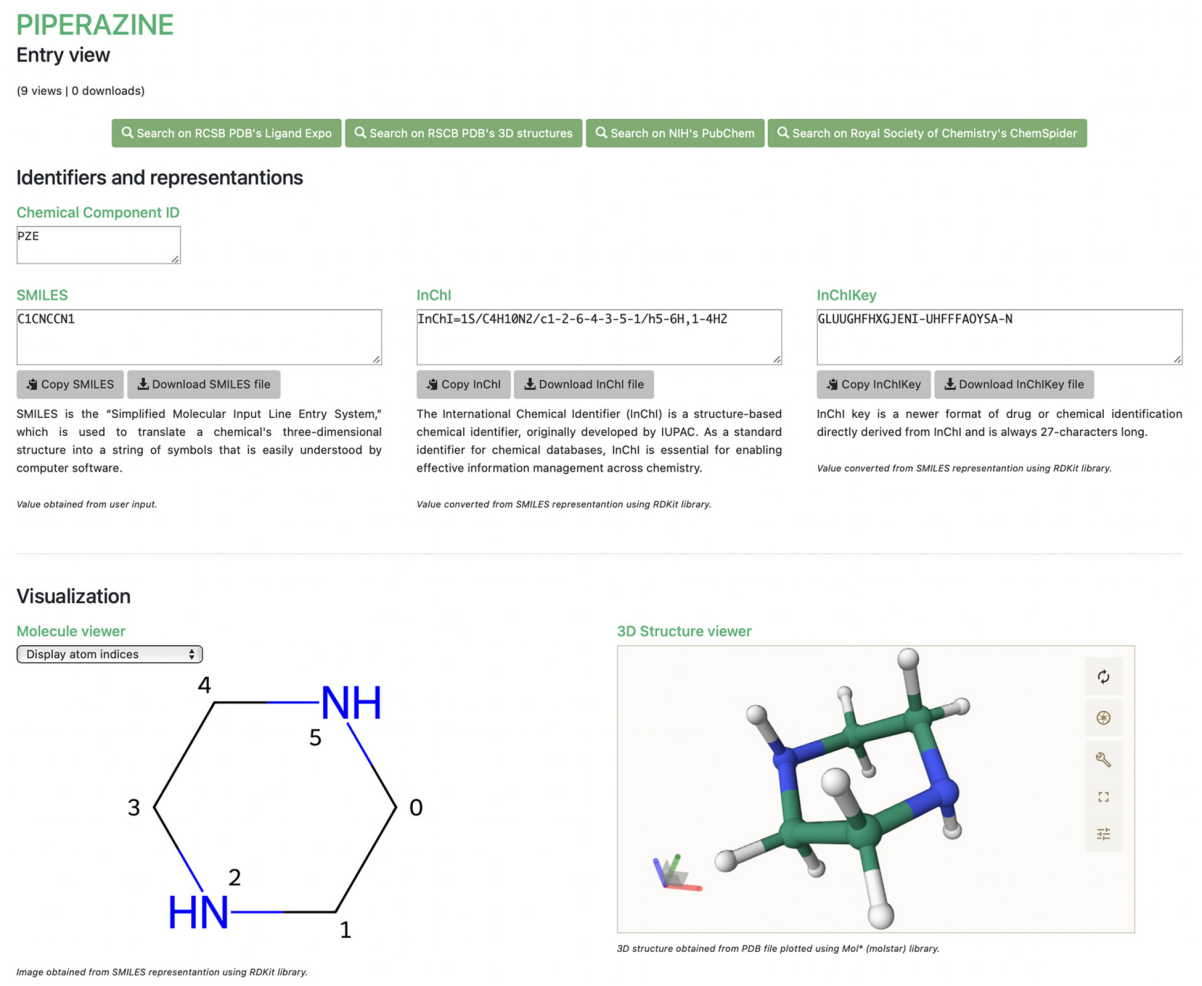
Visualization of an entry on the ACPYPE pages.

The uploaded files and pre-calculated results can be browsed and visualized (Fig. 1), with various information shown as well as links to AlphaFill (Hekkelman *et al.* 2023), which can help the user find biologically relevant leads for their molecule(s).

## 4 Conclusion

ACPYPE is one of the most popular programs for building small-molecule topologies for MD, especially for use with the Amber force field. After a decade of use, ACPYPE is robust and has now been updated with new features. ACPYPE is free of charge and its results can be obtained through multiple approaches.

## Supplementary Material

btad350_Supplementary_Data

## Data Availability

The data underlying this article are available in the article and in its online supplementary material or is accessible via the ACP YP E web server at https://bio2byte.be/acpype/.
