# Supplementary material for: The ACPYPE web server for small-molecule MD topology generation
Source: Bioinformatics. 2023 May 30;39(6):btad350. doi: 10.1093/bioinformatics/btad350 (PMC10264366; doi:10.1093/bioinformatics/btad350)
Supplement: btad350_Supplementary_Data [file btad350_supplementary_data.pdf]

Supplementary Material

The ACPYPE web server for small molecule MD topology generation

Luciano Kagami [1] , Alan W. Sousa da Silva [2] , Adrian Diaz[1,3], Wim Vranken [1,3]

[1] Interuniversity Institute of Bioinformatics in Brussels, VUB/ULB, Brussels, 1050, Belgium.
[2] Phenopolis Ltd & Moorfields Eye Hospital, London, UK
[3] Structural biology Brussels, Vrije Universiteit Brussel, Brussels, 1050, Belgium.

Table S1. Antechamber Errors List

Compound Name	Antechamber Error
radium	Unrecognized case-sensitive atomic symbol (RA).
titanium	Unrecognized case-sensitive atomic symbol (TI).
boric	No Gasteiger parameter for atom (ID: 1, Name: B, Type: DU).
vaborbactam	No Gasteiger parameter for atom (ID: 1, Name: B, Type: DU).
crisaborole	No Gasteiger parameter for atom (ID: 1, Name: B, Type: DU).
cacodylic	Unrecognized case-sensitive atomic symbol (AS).
oxophenarsine	Unrecognized case-sensitive atomic symbol (AS).
sulfarside	Unrecognized case-sensitive atomic symbol (AS).
tavaborole	No Gasteiger parameter for atom (ID: 1, Name: B, Type: DU).
carbarsone	Unrecognized case-sensitive atomic symbol (AS).
tryparsamide	Unrecognized case-sensitive atomic symbol (AS).
borofalan	No Gasteiger parameter for atom (ID: 0, Name: B, Type: DU).
thallous	Unrecognized case-sensitive atomic symbol (TL).
iron	Unrecognized case-sensitive atomic symbol (FE).
helium	No Gasteiger parameter for atom (ID: 0, Name: HE, Type: DU).
nitric	Cannot properly run sqm
zinc	Unrecognized case-sensitive atomic symbol (ZN).
xenon	Unrecognized case-sensitive atomic symbol (XE).

Table S2. SMILES Compound List and Status

Compound Name	Status
(2-benzhydryloxyethyl)diethyl-methylammonium	residue detected
(S)-nicardipine	residue detected

(S)-nitrendipine	residue detected
1-Octacosanol	success
2-(4-chlorphenoxy)-ethanol	success
4-dimethylaminophenol	success
5-aminolevulinic acid hexyl ester	success
abacavir	success
abaloparatide	more than one molecule detected
abametapir	success
abarelix	success
abemaciclib	success
abiraterone acetate	success
acadesine	success
acalabrutinib	success
acamprosate	success
acarbose	success
acebutolol	success
acecarbromal	success
aceclidine	success
aceclofenac	success
acedapsone	success
acediasulfone	success
acefylline	success
aceglatone	success
aceglutamide	more than one molecule detected
acemetacin	success
acenocoumarol	more than one molecule detected
acepromazine	success
aceprometazine	success
acetamidoeugenol	success
acetaminosalol	success
acetanilide	success

acetarsol	success
acetazolamide	success
acetic acid	success
acetohexamide	success
acetohydroxamic acid	success
acetophenazine	success
acetoxolone	success
acetrizoic acid	success
acetylcarnitine	success
acetylcholine	success
acetylcysteine	more than one molecule detected
acetyldigitoxin	success
acetyldigoxin	success
acetyldihydrocodeine	success
acetylleucine	more than one molecule detected
acetylmethadol	success
acetylpheneturide	success
acetylsalicylic acid	success
acexamic acid	success
acipimox	success
acitazanolast	success
acitretin	success
aclarubicin	success
aclatonium	success
aclidinium bromide	more than one molecule detected
acotiamide	success
acrivastine	success
actarit	success
actinoquinol	success
acyclovir	success
adamexine	success

adapalene	success
adefovir dipivoxil	success
ademetionine	more than one molecule detected
adenine	success
adenosine	success
adenosine	time exceeded
adenosine phosphate	success
adicillin	success
adinazolam	success
adiphenine	success
adipic acid	success
adipiodone	success
adrafinil	success
adrenalone	success
afamelanotide	time exceeded
afatinib	success
afloqualone	success
agomelatine	success
ajmaline	success
alacepril	more than one molecule detected
alanine	success
alanyl glutamine	more than one molecule detected
alatrofloxacin	success
albendazole	success
albendazole sulfoxide	success
albiglutide	more than one molecule detected
albutoin	success
alcaftadine	success
alclofenac	success
alclometasone dipropionate	success

alcuronium	more than one molecule detected
aldesulfone	success
aldosterone	success
alectinib	success
alendronic acid	success
alexidine	success
alfacalcidol	success
alfadolone	time exceeded
alfaxalone	success
alfentanil	success
alfuzosin	success
algeldrate	success
algestone	time exceeded
alibendol	success
alimemazine	success
aliskiren	success
alitretinoin	success
alizapride	success
alkofanone	success
allantoin	success
allobarbital	success
alloclamide	success
allomethadione	success
allopurinol	success
allylestrenol	success
almecillin	success
alminoprofen	success
almitrine	success
almotriptan	success
alogliptin	success
alosetron	success
alpelisib	success

alpha-Ergocryptine	success
alpha-tocopherol	time exceeded
alphaprodine	success
alpidem	success
alpiropride	success
alprazolam	success
alprenolol	success
alprostadil	success
alsactide	time exceeded
altizide	success
altretamine	success
altropane	success
aluminium oxide	success
alverine	success
alvimopan	success
amanozine	success
amantadine	success
ambazone	success
ambenonium	success
ambrisentan	success
ambroxol	success
ambucetamide	success
ambuside	success
ambutonium	success
amcinonide	success
amenamevir	success
amezinium metilsulfate	more than one molecule detected
amfecloral	success
amfetamine	success
amfetaminil	success
amidefrine	success
amifampridine	success

amifostine	success
amikacin	success
amiloride	success
amiloxate	success
aminaphthone	success
amineptine	success
aminitrozole	success
amino(diphenylhydantoin) valeric acid	success
aminoacridine	success
aminobenzoic acid	success
aminobutyric acid	success
aminocaproic acid	success
aminoglutethimide	success
aminohippuric acid	success
aminohydroxybutyric acid	success
aminolevulinic acid	success
aminomethylbenzoic acid	success
aminometradine	success
aminophenazone	success
aminopicoline	success
aminopromazine	success
aminopropylone	success
aminopterin	more than one molecule detected
aminoquinuride	success
aminorex	success
aminosalicylic acid	success
aminosalicylic acid hydrazide	success
aminoxytriphene	success
amiodarone	success
amiphenazole	success
amisometradine	success
amisulpride	success

amitriptyline	success
amitriptylinoxide	more than one molecule detected
amixetrine	success
amlexanox	success
amlodipine	success
ammonia	success
AMMONIA N-13	success
amobarbital	success
amocarzine	success
amodiaquine	success
amolanone	success
amorolfine	success
amoscanate	success
amosulalol	success
amoxapine	success
amoxicillin	success
amperozide	success
amphenidone	success
amphotalide	success
amphotericin	intramolecular bonds
ampicillin	success
ampiroxicam	success
amprenavir	success
amprotropine	success
amrinone	success
amrubicin	success
amsacrine	success
amtolmetin guacil	success
amylocaine	success
anagestone acetate	success
anagliptin	success
anagrelide	success

anastrozole	success
anazolene	success
ancitabine	success
androisoxazole	success
androstanolone	success
androstenediol	success
androstenedione	success
anecortave	success
anethole trithione	success
angiotensin II	more than one molecule detected
angiotensinamide	more than one molecule detected
anhydrovinblastine	success
anidulafungin	intramolecular bonds
anileridine	success
aniracetam	success
anisindione	success
antazoline	success
antimony	intramolecular bonds
antrafenine	success
apalcillin	success
apalutamide	success
apatinib	success
apixaban	success
apomorphine	success
apraclonidine	success
apremilast	success
aprepitant	success
aprindine	success
aprobarbital	success
apronal	success
aranidipine	success

arbekacin	success
arbutamine	success
arbutin	success
arformoterol	success
argatroban	success
arginine hydrochloride	success
aripiprazole	success
aripiprazole	time exceeded
armodafinil	success
arotinolol	success
arsanilic acid	success
arsenic trioxide	success
arsthinol	success
artemether	success
artemisinin	success
artemotil	success
artenimol	success
arterolane	success
artesunate	success
articaine	success
artisone acetate	success
ascorbic acid	success
asenapine	success
asparagine	success
aspartic acid	success
aspoxicillin	success
astemizole	success
astromicin	success
asulacrine	success
asunaprevir	success
ataluren	success
atazanavir	success
atenolol	success

atomoxetine	success
atorvastatin	success
atosiban	success
atovaquone	success
atracurium	intramolecular bonds
atrolactamide	success
atropine	success
atropine oxyde	more than one molecule detected
avanafil	success
avapritinib	success
avatrombopag	success
avibactam	success
avobenzone	success
axitinib	success
azacitidine	success
azacyclonol	success
azanidazole	success
azapetine	success
azapropazone	success
azaribine	success
azasetron	success
azatadine	success
azathioprine	success
azelaic acid	success
azelastine	success
azelnidipine	success
azidamfenicol	success
azidocillin	success
azilsartan	time exceeded
azimilide	success
azintamide	success
azithromycin	success

azlocillin	success
azosemide	success
aztreonam	success
bacampicillin	success
baclofen	success
bagrosin	success
baicalin	success
balofloxacin	success
baloxavir marboxil	success
balsalazide	success
bambuterol	success
bamethan	success
bamifylline	success
bamipine	success
barbital	success
baricitinib	success
barnidipine	success
bazedoxifene	success
beclabuvir	success
beclamide	success
beclobrate	success
beclometasone	time exceeded
bedaquiline	success
befunolol	success
bekanamycin	success
belinostat	success
belotecan	success
bemegride	success
bemetizide	success
bemotrizinol	success
bempedoic acid	success
benactyzine	success
benazepril	success

bencyclane	success
bendamustine	success
bendazac	success
bendazol	success
bendroflumethiazide	success
benexate	success
benfluorex	success
benfotiamine	success
benidipine	success
benmoxin	success
benorilate	success
benoxaprofen	success
benperidol	success
benproperine	success
benserazide	success
bentazepam	success
bentiromide	success
benzalamide	success
benzalkonium	success
benzamidosalicylate	success
benzarone	success
benzatropine	success
benzbromarone	success
benzestrol	success
benzethonium	success
benzilonium	success
benziodarone	success
benznidazole	success
benzocaine	success
benzoctamine	success
benzoic acid	success
benzoin	success
benzomethamine	success

benzoxonium	time exceeded
benzoyl peroxide	success
benzphetamine	success
benzpiperylone	success
benzquercin	success
benzquinamide	success
benzthiazide	success
benzydamine	success
benzyl alcohol	success
benzyl benzoate	success
benzyl nicotinate	success
benzylhydrochlorothiazide	success
benzylpenicillin	success
benzylsulfamide	success
benzylthiouracil	success
bephenium	success
bepotastine	success
bepridil	success
beraprost	success
bergapten	success
bermoprofen	success
berotralstat	success
besifloxacin	success
beta-Ergocryptine	success
betacarotene	success
betadex	success
betahistine	success
betaine	success
betamethasone	success
betamethasone	time exceeded
betamethasone	time exceeded
betamethasone	time exceeded
betamethasone	time exceeded

betamethasone acetate	success
betamethasone benzoate	success
betamethasone valerate	success
betamipron	success
betanaphthol	success
betanidine	success
betaxolol	success
betazole	success
bethanechol	success
betiatide	success
betoxycaine	success
betrixaban	success
bevantolol	success
bevonium	success
bexarotene	success
bezafibrate	success
bezitramide	success
bialamicol	success
biapenem	more than one molecule detected
bibenzonium	success
bibrocathol	success
bicalutamide	success
bictegravir	success
bidisomide	success
bietamiverine	success
bietaserpine	success
bifemelane	success
bifluranol	success
bifonazole	success
bilastine	success
bimatoprost	success
binifibrate	success

binimetinib	success
bioallethrin	success
biotin	success
biperiden	success
biphenylol	success
biriperone	success
bisacodyl	success
bisantrene	success
bisaramil	success
bisbentiamine	success
bisdequalinium chloride	time exceeded
bismuth camphocarbonate	success
bismuth subgallate	success
bismuth subsalicylate	success
bisoctrizole	success
bisoprolol	success
bisorcic	success
bisoxatin	success
bisoxatin acetate	success
bisulepine	success
bithionol	success
bitolterol	success
bitoscanate	success
bivalirudin	more than one molecule detected
bivalirudin	time exceeded
blonanserin	success
boceprevir	success
bolandiol dipropionate	success
bopindolol	success
boric	Antechamber failed
bornaprine	success
borofalan	Antechamber failed

bortezomib	intramolecular bonds
bosentan	success
bosutinib	success
brallobarbital	success
bremelanotide	intramolecular bonds
bretylium	success
brexanolone	success
brexpiprazole	success
brigatinib	success
brilliant Blue G	success
brilliant green	more than one molecule detected
brimonidine	success
brinzolamide	success
brivaracetam	success
brivudine	success
brodimoprim	success
bromazepam	success
bromazine	success
bromebric acid	success
bromfenac	success
bromhexine	success
bromindione	success
bromisoval	success
bromochlorosalicylanilide	success
bromocriptine	success
bromoform	success
bromopride	success
bromperidol	success
brompheniramine	success
bropirimine	success
brotizolam	success
brovanexine	success

brovincamine	success
broxuridine	success
broxyquinoline	success
bucetin	success
bucillamine	more than one molecule detected
bucladesine	success
buclizine	success
buclosamide	success
bucloxic acid	success
bucolome	success
bucricaine	success
bucumolol	success
budesonide	success
budipine	success
budralazine	success
bufeniode	success
bufetolol	success
bufexamac	success
buflomedil	success
bufogenin	success
buformin	success
bufuralol	success
bulaquine	success
bumadizone	success
bumetanide	success
bunaftine	success
bunamiodyl	intramolecular bonds
bunazosin	success
bunitrolol	success
buphenine	success
bupivacaine	success
bupranolol	success

buprenorphine	success
bupropion	success
buramate	success
buserelin	success
buspirone	success
busulfan	success
butacaine	success
butacetin	success
butalamine	success
butalbital	success
butamben	success
butamirate	success
butanilicaine	success
butaperazine	success
butaverine	success
butazolamide	success
butazopyridine	success
butedronic acid	success
butenafine	success
butetamate	success
butethamine	success
buthalital	success
butibufen	success
butidrine	success
butinoline	success
butizide	success
butobarbital	success
butobendine	success
butoconazole	success
butoctamide	success
butofilolol	success
butorphanol	success
butoxycaine	success

butriptyline	success
butropium	success
butylphthalide	success
buzepide metiodide	more than one molecule detected
cabazitaxel	success
cabergoline	success
cabotegravir	success
cabozantinib	success
cacodylic	Antechamber failed
cadralazine	success
cafaminol	success
cafedrine	success
caffeine	success
calcifediol	success
calcipotriene	success
calcitonin	time exceeded
calcitonin (pork natural)	success
calcitonin human	success
calcitriol	success
calcium pantothenate	success
calusterone	success
camazepam	success
camostat	success
camphor	success
camylofin	success
canagliflozin	success
candesartan	time exceeded
candicidin	intramolecular bonds
canfosfamide	success
cangrelor	success
cannabidiol	success
canrenone	success

capecitabine	success
capmatinib	success
capobenic acid	success
capsaicin	success
captodiame	success
captopril	success
capuride	success
caramiphen	success
carazolol	success
carbachol	success
carbamazepine	success
carbamide	success
carbarsone	Antechamber failed
carbaryl	success
carbazochrome	success
carbenicillin	success
carbenoxolone	success
carbetocin	success
carbidopa	success
carbifene	success
carbimazole	success
carbinoxamine	success
carbocisteine	success
carbocloral	success
carbocromen	success
carbocysteine-lysine	more than one molecule detected
carbon dioxide	success
carbon tetrachloride	success
carboprost	success
carboquone	success
carbromal	success
carbubarb	success

carbutamide	success
carbuterol	success
carfecillin	success
carfenazine	success
carfilzomib	success
carfimate	success
carglumic acid	more than one molecule detected
cargutocin	success
carindacillin	success
cariprazine	success
carisbamate	success
carisoprodol	success
carmofur	success
carmustine	success
caroverine	success
caroxazone	success
carperitide	time exceeded
carpipramine	success
carprofen	success
carpronium	success
carsalam	success
carteolol	success
carubicin	success
carumonam	success
carvedilol	success
casopitant	success
caspofungin	intramolecular bonds
cathine	success
cefacetrile	success
cefaclor	success
cefadroxil	success
cefalexin	success

cefalexin	time exceeded
cefaloglycin	success
cefaloridine	more than one molecule detected
cefalotin	success
cefamandole	success
cefamandole nafate	success
cefapirin	success
cefathiamidine	success
cefatrizine	success
cefazedone	success
cefazolin	success
cefbuperazone	success
cefcapene	success
cefcapene	time exceeded
cefdinir	success
cefditoren	time exceeded
cefepime	success
cefetamet	success
cefetamet pivoxil	success
cefiderocol	success
cefixime	success
cefmenoxime	success
cefmetazole	success
cefminox	success
cefodizime	success
cefonicid	success
cefoperazone	success
ceforanide	success
cefoselis	more than one molecule detected
cefotaxime	success
cefotetan	success

cefotiam	success
cefotiam	time exceeded
cefoxitin	success
cefozopran	more than one molecule detected
cefpimizole	success
cefpiramide	success
cefpirome	more than one molecule detected
cefpodoxime	time exceeded
cefprozil	success
cefradine	success
cefroxadine	success
cefsulodin	time exceeded
ceftaroline fosamil	intramolecular bonds
ceftazidime	time exceeded
cefteram	success
cefteram	time exceeded
ceftezole	success
ceftibuten	time exceeded
ceftizoxime	more than one molecule detected
ceftobiprole	time exceeded
ceftolozane	time exceeded
ceftriaxone	success
cefuroxime	success
cefuroxime	time exceeded
cefuroxime axetil	success
cefuzonam	success
celecoxib	success
celiprolol	success
cenobamate	success
cepharanthine	success
ceritinib	success

cerivastatin	success
cerulenin	success
ceruletide	success
cetalkonium chloride	time exceeded
cetamolol	success
cethexonium	success
cetiedil	success
cetilistat	success
cetirizine	success
cetotiamine	success
cetoxime	success
cetraxate	success
cetrimonium	success
cetrorelix	success
cetyl alcohol	success
cetylpyridinium	more than one molecule detected
cevimeline	success
chenodiol	success
chidamide	success
chloral hydrate	success
chloralodol	success
chloralose	success
chlorambucil	success
chloramphenicol	success
chloramphenicol	time exceeded
chloramphenicol palmitate	more than one molecule detected
chlorazanil	success
chlorbenzoxamine	success
chlorbetamide	success
chlorcyclizine	success
chlordiazepoxide	success

chlorfenethazine	success
chlorhexidine	success
chlorisondamine	success
chlormadinone	success
chlormezanone	success
chlormidazole	success
chlornaphazine	success
chlorobutanol	success
chloroform	success
chloroprednisone	success
chloroprocaine	success
chloropyramine	success
chloropyrilene	success
chloroquine	success
chlorothiazide	success
chlorotrianisene	success
chloroxine	success
chloroxylenol	success
chlorozotocin	success
chlorphenamine	success
chlorphenesin carbamate	success
chlorphenoxamine	success
chlorphentermine	success
chlorproethazine	success
chlorproguanil	success
chlorpromazine	success
chlorpropamide	success
chlorprothixene	success
chlorquinaldol	success
chlortalidone	success
chlortetracycline	success
chlorthenoxazine	success
chlorzoxazone	success

cholic acid	success
choline	success
choline alfoscerate	more than one molecule detected
Choline C-11	more than one molecule detected
choline fenofibrate	success
chromic	intramolecular bonds
chromocarb	success
cianidanol	success
cibenzoline	success
cicaprost	success
ciclesonide	success
cicletanine	success
ciclobendazole	success
ciclonicate	success
ciclonium	success
ciclopirox	success
cicloprolol	success
ciclosidomine	success
ciclosporin	intramolecular bonds
cicrotoic acid	success
cidofovir	success
cilastatin	success
cilazapril	success
cilnidipine	success
cilostazol	success
cimetidine	success
cimetropium	success
cinacalcet	success
cinalukast	success
cinametic acid	success
cinchocaine	success

cinchophen	success
cineole	success
cinepazet	success
cinepazide	success
cinitapride	success
cinmetacin	success
cinnamaverine	success
cinnarizine	success
cinnoxicam	success
cinolazepam	success
cinoxacin	success
cinromide	success
cioteronel	success
ciprofibrate	success
ciprofloxacin	success
ciramadol	success
cisapride	success
cisatracurium	success
citalopram	success
citicoline	success
citiolone	success
citric acid	success
citrulline	success
cladribine	success
clanobutin	success
clarithromycin	success
clascoterone	success
clavulanic acid	success
clebopride	success
clefamide	success
clemastine	success
clemizole	success
clenbuterol	success

clevidipine	success
clevudine	success
clidanac	success
clidinium	success
climbazole	success
clinafloxacin	success
clindamycin	success
clindamycin	time exceeded
clindamycin phosphate	success
clinofibrate	success
clioquinol	success
clobazam	success
clobenoside	success
clobenzepam	success
clobenzorex	success
clobenztropine	success
clobetasol propionate	success
clobetasone butyrate	success
clobutinol	success
clobuzarit	success
clocapramine	success
clocinizine	success
clocortolone pivalate	success
clodantoin	success
clodronic acid	success
clofarabine	success
clofazimine	success
clofedanol	success
clofenamide	success
clofenotane	success
clofibrate	success
clofibric acid	success
clofibride	success

clofoctol	success
cloforex	success
clomacran	success
clometacin	success
clomethiazole	success
clometocillin	success
clomifene	success
clomipramine	success
clomocycline	success
clonazepam	success
clonidine	success
clonitrate	success
clonixin	success
clopamide	success
clopenthixol	success
cloperastine	success
clopidogrel	success
clopirac	success
cloprednol	success
cloranolol	success
clorazepate	success
clorexolone	success
cloricromen	success
cloridarol	success
clorindione	success
clorotepine	success
clorprenaline	success
clortermine	success
clostebol	success
clostebol acetate	success
clotiapine	success
clotiazepam	success
clotrimazole	success

cloxacillin	success
cloxazolam	success
cloxiquine	success
cloxotestosterone	success
clozapine	success
cobicistat	success
cobimetinib	success
cocaine	success
codeine	success
colchicine	success
colecalciferol	success
colforsin daropate	success
colfosceril palmitate	time exceeded
conivaptan	success
copanlisib	success
copper	time exceeded
corbadrine	success
corticorelin	more than one molecule detected
corticorelin	time exceeded
corticotropin	more than one molecule detected
cortisone acetate	success
cortivazol	success
cotinine	success
coumarin	success
coumetarol	success
creatine	success
creatinolfosfate	success
cresatin	success
cridanimod	success
crisaborole	Antechamber failed
crizotinib	success

croconazole	success
cromoglicic	time exceeded
cropropamide	success
crotamiton	success
crotetamide	success
cupric	intramolecular bonds
cupric oxide	success
cyamemazine	success
cyclacillin	success
cyclandelate	success
cyclarbamate	success
cyclizine	success
cyclobarbital	success
cyclobenzaprine	success
cyclobutyrol	success
cyclocoumarol	success
cyclodrine	success
cyclofenil	success
cycloguanil	success
cyclomethycaine	success
cyclopentamine	success
cyclopenthiazide	success
cyclopentobarbital	success
cyclopentolate	success
cyclophosphamide	success
cycloserine	success
cyclothiazide	success
cyclovalone	success
cycloxilic acid	success
cycrimine	success
cyfluthrin	success
cymarin	success
cynarine	success

cypermethrin	success
cyprodenate	success
cyproheptadine	success
cyproterone acetate	success
cysteine	success
cystine	success
cytarabine	success
cytarabine	time exceeded
cytisine	success
cyverine	success
dabigatran	time exceeded
dabrafenib	success
dacarbazine	success
dacisteine	success
daclatasvir	success
dacomitinib	success
dactinomycin	success
dalbavancin	time exceeded
dalfopristin	time exceeded
danazol	success
dantrolene	success
dantron	success
dapagliflozin	success
dapiprazole	success
dapoxetine	success
daprodustat	success
dapsone	success
daptomycin	time exceeded
darifenacin	success
darolutamide	success
darunavir	success
dasabuvir	success
dasatinib	success

dasiglucagon	more than one molecule detected
daunorubicin	success
daunorubicinol	success
davercin	success
deanol	success
debrisoquine	success
decamethonium	success
decamethoxine	success
decamethrin	success
decimemide	success
decitabine	success
deferasirox	success
deferiprone	success
deferoxamine	success
deflazacort	success
degarelix	time exceeded
dehydrocholic acid	success
delafloxacin	success
delamanid	success
delapril	success
delavirdine	success
delgocitinib	success
delmadinone acetate	success
delmopinol	success
delorazepam	success
demecarium	success
demeclocycline	success
demecolcine	success
demegestone	success
demexiptiline	success
demoxytocin	success
denaverine	success

denopamine	success
deoxycholic acid	success
depreotide	time exceeded
deptropine	success
dequalinium	success
desalkylflurazepam	success
desaspidin	success
deserpidine	success
desflurane	success
desipramine	success
deslanoside	success
desloratadine	success
desmopressin	success
desogestrel	success
desomorphine	success
desonide	success
desoximetasone	success
desoxycorticosterone	time exceeded
desoxycorticosterone acetate	success
desoxycortone	success
desoxymycin	success
desvenlafaxine	success
detajmium	success
deutetrabenazine	success
dexamethasone	success
dexamethasone	time exceeded
dexamethasone	time exceeded
dexamethasone	time exceeded
dexamethasone	time exceeded
dexamethasone acetate	success
dexamethasone phosphate	success
dexamfetamine	success
dexbrompheniramine	success

dexchlorpheniramine	success
dexetimide	success
dexfenfluramine	success
dexfosfoserine	success
dexibuprofen	success
dexketoprofen	success
dexlansoprazole	success
dexloxiglumide	success
dexmedetomidine	success
dexmethylphenidate	success
dexniguldipine	success
dexpanthenol	success
dexrabeprazole	success
dexrazoxane	success
dextrofemine	success
dextromethorphan	success
dextromoramide	success
dextropropoxyphene	success
dextrothyroxine	success
dezocine	success
diacerein	success
diacetazotol	success
diacetyl	time exceeded
diamorphine	success
diampromide	success
diathymosulfone	time exceeded
diatrizoate	success
diazepam	success
diaziquone	success
diazolidinylurea	success
diazoxide	success
dibekacin	success
dibenzepin	success

dibromol	success
dibromotyrosine	success
dibrompropamidine	success
dibunate	success
dibutylphthalate	success
dibutylsuccinate	success
dichlorisone	success
dichloroacetic acid	success
dichlorobenzyl alcohol	success
dichlorophen	success
dichlorophenarsine	time exceeded
dichlorphenamide	success
diclofenac	success
dicloxacillin	success
dicoumarol	success
dicycloverine	success
didanosine	success
dienestrol	success
dienogest	success
diethadione	success
diethazine	success
diethyl ether	success
diethylamino	time exceeded
diethylaminoethoxyhexestrol	success
diethylcarbamazine	success
diethylpropion	success
diethylstilbestrol	success
diethyltoluamide	success
difebarbamate	success
difemerine	success
difemetorex	success
difenoxin	success
difenpiramide	success

difetarsone	time exceeded
diflorasone diacetate	success
diflucortolone	success
diflunisal	success
difluprednate	success
digitalin	success
digitoxin	time exceeded
digoxin	success
dihexyverine	success
dihydralazine	success
dihydrocodeine	success
dihydroemetine	success
dihydroergocornine	success
dihydroergocristine	success
dihydroergocryptine	success
dihydroergotamine	success
dihydrostreptomycin	success
dihydrotachysterol	success
diiodohydroxypropane	intramolecular bonds
diiodohydroxyquinoline	success
diiodotyrosine	success
diisopromine	success
dilazep	success
dilevalol	success
diloxanide furoate	success
diltiazem	success
dimantine	success
dimazole	success
dimecrotic acid	success
dimefline	success
dimemorfan	success
dimenoxadol	success
dimepheptanol	success

dimercaprol	success
dimetacrine	success
dimethazan	success
dimethisterone	success
dimethocaine	success
dimethoxanate	success
dimethyl fumarate	success
dimethyl sulfoxide	success
dimethylaminopropionylphenothiazine	success
dimethylcarbate	success
dimethylphthalate	success
dimethylthiambutene	success
dimethyltubocurarinium	success
dimetindene	success
dimetofrine	success
dimetotiazine	success
dimevamide	success
dimorpholamine	success
dimyristoylphosphatidylcholine	success
dimyristoylphosphatidylglycerol	success
dinoprost	success
dinoprostone	success
diosmetin	success
diosmin	success
dioxadrol	success
dioxaphetyl butyrate	success
dioxethedrin	success
dioxybenzone	success
dioxyline	success
diperodon	success
diphemanil	success
diphenadione	success
diphenan	success

diphenhydramine	success
diphenidol	success
diphenoxylate	success
diphenylpyraline	success
dipipanone	success
dipiperonylaminoethanol	success
dipiproverine	success
dipivefrine	success
diprafenone	success
diprophylline	success
dipyridamole	success
dipyrithione	success
dipyrocetyl	success
diquafosol	success
dirithromycin	success
diseptal B	success
disofenin	success
disopyramide	success
distigmine	success
disufenton	success
disulfamide	success
disulfiram	success
ditazole	success
dithiazanine	intramolecular bonds
dithranol	success
ditiocarb	success
dixanthogen	success
dixyrazine	success
DL-Alanine	success
dobutamine	success
docarpamine	success
docetaxel	success
doconexent	success

docosanoic acid	success
docosanol	success
docusate sodium	success
dodecanoic acid	success
dodeclonium bromide	more than one molecule detected
dofetilide	success
dolasetron	success
dolutegravir	success
domiodol	success
domiphen	success
domitroban	success
domperidone	success
donepezil	success
dopamine	success
dopexamine	success
doravirine	success
doripenem	success
dorzolamide	success
dosulepin	success
dotinurad	success
doxacurium	time exceeded
doxapram	success
doxazosin	success
doxefazepam	success
doxepin	success
doxercalciferol	success
doxifluridine	success
doxofylline	success
doxorubicin	success
doxycycline	success
doxylamine	success
drofenine	success

drometrizole	success
dronabinol	success
dronedarone	success
droperidol	success
droprenilamine	success
dropropizine	success
drospirenone	success
drostanolone	time exceeded
drotaverine	success
drotebanol	success
droxicam	success
droxidopa	success
droxypropine	success
duloxetine	success
dutasteride	success
duvelisib	success
dyclonine	success
dydrogesterone	success
ebastine	success
eberconazole	success
ebrotidine	success
eburnamonine	success
ecabet	success
ecamsule	success
echothiophate	success
econazole	success
ectylurea	success
edaravone	success
edatrexate	more than one molecule detected
edetic acid	success
edoxaban	success
edoxudine	success

edrophonium	success
efaproxiral	success
efavirenz	success
efinaconazole	success
eflornithine	success
efloxate	success
efonidipine	success
egualen	success
eicosapentaenoic	time exceeded
EIDD-1931	success
elagolix	success
elbasvir	success
elcatonin	time exceeded
elcometrine	success
eldecalcitol	success
eledoisin	success
eletriptan	success
elexacaftor	success
eliglustat	success
elliptinium	more than one molecule detected
elobixibat	time exceeded
eltanolone	success
eltrombopag	success
eluxadoline	success
elvitegravir	success
embramine	success
emedastine	success
emepronium	success
emetine	success
emorfazone	success
empagliflozin	success
emtricitabine	success

emylcamate	success
enalapril	more than one molecule detected
enalaprilat	more than one molecule detected
enallylpropymal	success
enarodustat	success
enasidenib	success
enbucrilate	success
encainide	success
encorafenib	success
endralazine	success
enfenamic acid	success
enflurane	success
enfuvirtide	time exceeded
enilconazole	success
enocitabine	success
enoxacin	success
enoximone	success
enoxolone	time exceeded
enprofylline	success
enprostil	success
ensulizole	success
entacapone	success
entecavir	success
entrectinib	success
enviomycin	more than one molecule detected
enzacamene	success
enzalutamide	success
eosin	success
epalrestat	success
epanolol	success
eperisone	success

epervudine	success
ephedrine	success
epicillin	success
epicriptine	success
epimestrol	success
epinastine	success
epinephrine	success
epirizole	success
epirubicin	success
epitiostanol	success
epitizide	success
eplerenone	success
epomediol	success
epoprostenol	success
eprazinone	success
eprosartan	success
eprozinol	success
eptazocine	success
eptifibatide	success
eravacycline	time exceeded
erdafitinib	success
erdosteine	success
ergocalciferol	success
ergometrine	more than one molecule detected
ergotamine	success
eribulin	success
eritoran	success
eritrityl tetranitrate	more than one molecule detected
erlotinib	success
ertapenem	success
ertugliflozin	success

erythromycin	success
erythromycin	time exceeded
erythromycin	time exceeded
erythromycin	time exceeded
esatenolol	success
escitalopram	success
eseridine	success
esflurbiprofen	success
esketamine	success
eslicarbazepine acetate	success
esmolol	success
esomeprazole	success
estazolam	success
estetrol	success
estradiol	success
estradiol	time exceeded
estradiol	time exceeded
estradiol	time exceeded
estradiol acetate	success
estradiol benzoate	success
estradiol cypionate	success
estradiol dipropionate	success
estradiol valerate	success
estramustine	success
estramustine phosphate	success
estriol	success
estriol succinate	success
estrone	success
estrone sulphate	success
eszopiclone	success
etafedrine	success
etafenone	success
etallobarbital	success

etamiphylline	success
etamivan	success
etaqualone	success
etebenecid	success
etelcalcetide	success
eterobarb	success
etersalate	success
ethacizine	success
ethacridine	success
ethacrynic acid	success
ethadione	success
ethambutol	success
ethanol	success
ethaverine	success
ethchlorvynol	success
ethenzamide	success
ethinamate	success
ethinylestradiol	success
ethinylestradiol sulfonate	success
ethionamide	success
ethisterone	success
ethoheptazine	success
ethosuximide	success
ethotoin	success
ethoxzolamide	success
ethyl biscoumacetate	success
ethyl chloride	success
ethyl dibunate	success
ethyl hydroxybenzoate	success
ethyl loflazepate	success
ethylestrenol	success
ethylhydrocupreine	success
ethylmethylthiambutene	success

ethylmorphine	success
ethylnorepinephrine	success
etidocaine	success
etidronic acid	success
etifelmine	success
etifoxine	success
etilamfetamine	success
etilefrine	success
etilefrine pivalate	success
etilevodopa	success
etimicin	success
etiprednol dicloacetate	success
etiroxate	success
etizolam	success
etodolac	success
etodroxizine	success
etofamide	success
etofenamate	success
etofibrate	success
etofylline	success
etofylline	time exceeded
etofylline nicotinate	success
etoglucid	success
etohexadiol	success
etomidate	success
etomidoline	success
etonogestrel	success
etoperidone	success
etoposide	success
etoposide phosphate	success
etoricoxib	success
etoxadrol	success
etoxazene	success

etozolin	success
etravirine	success
etretinate	success
etryptamine	success
etybenzatropine	success
etymemazine	success
etynodiol	success
eucatropine	success
eugenol	success
evans	time exceeded
everolimus	intramolecular bonds
evocalcet	success
evogliptin	success
exalamide	success
exatecan	success
exemestane	success
exenatide	time exceeded
exiproben	success
exisulind	success
ezetimibe	success
fabomotizole	success
fadrozole	success
falecalcitriol	success
famciclovir	success
famotidine	success
fampridine	success
famprofazone	success
faropenem	success
faropenem medoxil	success
fasudil	success
favipiravir	success
fazadinium	success
febarbamate	success

febuprol	success
febuxostat	success
feclemine	success
feclobuzone	success
fedratinib	success
fedrilate	success
felbamate	success
felbinac	success
felodipine	success
felypressin	intramolecular bonds
femoxetine	success
fenadiazole	success
fenalamide	success
fenalcomine	success
fenamisal	success
fenbendazole	success
fenbenicillin	success
fenbufen	success
fenbutrazate	success
fencamfamin	success
fencibutirol	success
fenclofenac	success
fendiline	success
fendosal	success
fenethazine	success
fenetylline	success
fenfluramine	success
fenipentol	success
fenofibrate	success
fenoldopam	success
fenoprofen	success
fenoterol	success
fenoverine	success

fenoxazoline	success
fenoxedil	success
fenoxypropazine	success
fenozolone	success
fenpentadiol	success
fenpiprane	success
fenpiverinium	success
fenproporex	success
fenquizone	success
fenspiride	success
fentanyl	success
fentiazac	success
fenticlor	success
fenticonazole	success
fentonium	success
fenyramidol	success
fepradinol	success
feprazone	success
ferric	time exceeded
ferric	time exceeded
ferrous	time exceeded
fesoterodine	success
fexofenadine	success
fidaxomicin	success
filgotinib	success
fimasartan	success
finafloxacin	success
finasteride	success
fingolimod	success
fipexide	success
flavodic acid	success
flavoxate	success
flecainide	success

fleroxacin	success
flibanserin	success
floctafenine	success
flomoxef	success
flopropione	success
florantyrone	success
florbetaben F18	success
Florbetapir F-18	success
floredil	success
flortaucipir F 18	success
flosequinan	success
floxacillin	success
floxuridine	success
fluacizine	success
fluazacort	success
flubendazole	success
fluciclovine (18F)	success
fluclorolone	success
fluclorolone acetonide	success
fluconazole	success
flucytosine	success
fludarabine phosphate	success
fludeoxyglucose (18F)	success
fludiazepam	success
fludrocortisone acetate	success
fludroxycortide	success
flufenamic acid	success
fluindione	success
flumazenil	success
flumecinol	success
flumedroxone	success
flumedroxone acetate	success
flumequine	success

flumetasone	success
flumetasone	time exceeded
flumethiazide	success
flunarizine	success
flunisolide	success
flunitrazepam	success
flunoxaprofen	success
fluocinolone acetonide	success
fluocinonide	success
fluocortin	time exceeded
fluocortolone	success
fluoresone	success
fluorodopa (18F)	success
fluoroestradiol F 18	success
fluoroethylcholine (18F)	more than one molecule detected
fluorometholone	success
fluorometholone acetate	success
fluoromethylcholine (18F)	more than one molecule detected
fluorouracil	success
fluostigmine	success
fluoxetine	success
fluoxymesterone	success
flupentixol	success
fluperolone	success
fluphenazine	success
fluphenazine	time exceeded
fluphenazine enanthate	success
flupirtine	success
fluprednidene	success
fluprednidene acetate	success
fluprednisolone	success

flurazepam	success
flurbiprofen	success
flurbiprofen axetil	success
flurithromycin	success
flurotyl	success
fluroxene	success
flusalan	success
fluspirilene	success
flutamide	success
flutazolam	success
Flutemetamol (18F)	success
fluticasone	time exceeded
fluticasone propionate	success
flutoprazepam	success
flutrimazole	success
flutropium	success
fluvastatin	success
fluvoxamine	success
folescutol	success
folic acid	more than one molecule detected
fomepizole	success
fominoben	success
fomocaine	success
fondaparinux	success
formaldehyde	success
formebolone	success
formestane	success
formocortal	success
formoterol	success
formylsulfamethin	success
forodesine	success
fosamprenavir	success

fosaprepitant	success
foscarnet	success
fosdenopterin	success
fosfestrol	success
fosfluconazole	success
fosfomycin	success
fosfosal	success
fosinopril	success
fosnetupitant	success
fosphenytoin	success
fospropofol	success
fostamatinib	success
fostemsavir	success
fotemustine	success
framycetin	success
frovatriptan	success
fructose	success
ftivazide	success
fudosteine	success
fulvestrant	success
fumagillin	success
fumaric acid	success
furalazine	success
furaltadone	success
furamicid	success
furazidin	success
furazolidone	success
furfenorex	success
furonazide	success
furosemide	success
fursultiamine	success
furtrethonium	success
fusidic	time exceeded

fytic	intramolecular bonds
gabapentin	success
gabapentin enacarbil	success
gabexate	success
galactose	success
galantamine	success
gallamine	success
gallium	time exceeded
gallopamil	success
gamolenic	time exceeded
ganciclovir	success
ganirelix	time exceeded
garenoxacin	success
gatifloxacin	success
gefarnate	success
gefitinib	success
gemcitabine	success
gemeprost	success
gemfibrozil	success
gemifloxacin	success
gemigliptin	success
gentian violet	more than one molecule detected
gentisic acid	success
gepefrine	success
gepirone	success
gestodene	success
gestonorone	success
gestonorone	time exceeded
gestrinone	success
gilteritinib	success
gimeracil	success
gitoformate	success

gitoxin	success
glafenine	success
glasdegib	success
glecaprevir	success
glibenclamide	success
glibornuride	success
gliclazide	success
glimepiride	success
glipizide	success
gliquidone	success
glisentide	success
glisolamide	success
glisoxepide	success
glucagon	time exceeded
glucametacin	success
gluconic acid	success
gluconolactone	success
glucosamine	success
glucosaminylmuramyl	time exceeded
glucose	success
glucose-1-phosphate	success
glucosulfone	success
glucurolactone	success
glucuronamide	success
glutamic acid	success
glutamine	success
glutaral	success
glutathione	more than one molecule detected
glutethimide	success
glybuthiazol	success
glybuzole	success
glycerol	success

glycerol	time exceeded
glyceryl trinitrate	more than one molecule detected
glycerylphosphorylcholine	success
glycine	success
glycobiarsol	time exceeded
glycol salicylate	success
glycolic acid	success
glyconiazide	success
glycopyrronium bromide	more than one molecule detected
glycyclamide	success
glycyrrhizic	time exceeded
glyhexamide	success
glymidine	success
glypinamide	success
gonadorelin	success
goserelin	success
gossypol	success
granisetron	success
grazoprevir	success
grepafloxacin	success
griseofulvin	success
guabenxan	success
guacetisal	success
guaiacol	success
guaiacol	time exceeded
guaiacol benzoate	success
guaiacol carbonate	success
guaiapate	success
guaiazulen	success
guaifenesin	success
guaimesal	success

guamecycline	success
guanabenz	success
guanacline	success
guanadrel	success
guanazodine	success
guancidine	success
guanethidine	success
guanfacine	success
guanidine	success
guanoclor	success
guanoxabenz	success
guanoxan	success
gusperimus	success
halazepam	success
halcinonide	success
haletazole	success
halocarban	success
halofantrine	success
halometasone	success
haloperidol	success
haloperidol	time exceeded
halopredone acetate	success
haloprogin	success
halopropane	success
halothane	success
haloxazolam	success
helium	Antechamber failed
hematoporphyrin	success
hepronicate	success
heptabarb	success
heptaminol	success
heptobarbital	success
hesperetin	success

hetacillin	success
hexachlorophene	success
hexafluronium	success
hexamethonium	success
hexamidine	success
hexapropymate	success
hexcarbacholine	success
hexestrol	success
hexetidine	success
hexobarbital	success
hexobendine	success
hexocyclium	success
hexoprenaline	success
hexylcaine	success
hexyldecanoic acid	success
hexylresorcinol	success
hidrosmin	time exceeded
histamine	success
histapyrrodine	success
histidine	success
histrelin	time exceeded
homarylamine	success
homatropine	success
homocamfin	success
homochlorcyclizine	success
homofenazine	success
homonicotinic acid	success
homosalate	success
hopantenic acid	success
hycanthone	success
hydracarbazine	success
hydralazine	success
hydrochloric acid	success

hydrochlorothiazide	success
hydrocodone	success
hydrocortamate	success
hydrocortisone	success
hydrocortisone	time exceeded
hydrocortisone	time exceeded
hydrocortisone	time exceeded
hydrocortisone	time exceeded
hydrocortisone	time exceeded
hydrocortisone phosphate	success
hydrocortisone succinate	success
hydrocortisone valerate	success
hydroflumethiazide	success
hydrofluoric acid	success
hydrogen peroxide	success
hydromorphone	success
hydroquinidine	success
hydroquinine	success
hydroquinone	success
hydroxyamfetamine	success
hydroxycarbamide	success
hydroxychloroquine	success
hydroxyestrone diacetate	success
hydroxyethylpromethazine	success
hydroxymethylnicotinamide	success
hydroxypethidine	success
hydroxyprogesterone caproate	success
hydroxyproline	success
hydroxystilbamidine	success
hydroxytetracaine	success
hydroxyzine	success
hymecromone	success
hyoscyamine	success

ibacitabine	success
ibandronic acid	success
ibopamine	success
ibrexafungerp	more than one molecule detected
ibrutinib	success
ibudilast	success
ibufenac	success
ibuprofen	success
ibuprofen guaiacol ester	success
ibuprofen piconol	success
ibuproxam	success
ibutilide	success
icatibant	more than one molecule detected
iclaprim	success
icosapent	time exceeded
icotinib	success
idanpramine	success
idarubicin	success
idebenone	success
idelalisib	success
idoxuridine	success
idrocilamide	success
ifenprodil	success
ifosfamide	success
iguratimod	success
ilaprazole	success
iloperidone	success
iloprost	success
imatinib	success
imiclopazine	success
imidafenacin	success

imidapril	more than one molecule detected
imipenem	success
imipramine	success
imipramine oxide	more than one molecule detected
imiquimod	success
imolamine	success
improsulfan	success
imrecoxib	success
inaperisone	success
incadronic acid	success
indacaterol	success
indalpine	success
indanazoline	success
indapamide	success
indecainide	success
indeloxazine	success
indigo carmine	success
indinavir	success
indisetron	success
indobufen	success
indocyanine green	time exceeded
indomethacin	success
indoprofen	success
indoramin	success
infigratinib	success
ingenol mebutate	success
inosine	success
inositol	success
inositol	time exceeded
inqovi	success
intoplicine	success

iobenguane (123I)	success
iobenguane (131I)	success
iobenzamic acid	success
iobitridol	success
iocarmic	time exceeded
iocetamic acid	success
iodamide	success
iodinated glycerol	success
iodine	intramolecular bonds
iodixanol	success
iodocholesterol	time exceeded
iodoform	success
iodohippurate sodium I125	success
iodohippurate sodium I131	success
iodol	success
iodothiouracil	success
iodoxamic	time exceeded
Iofetamine (123I)	success
Ioflupane I-123	success
ioglicic	intramolecular bonds
ioglycamic acid	success
iohexol	success
iomazenil (123I)	success
iomeglamic acid	success
iomeprol	success
iopamidol	more than one molecule detected
iopanoic acid	success
iopentol	success
iophenoic acid	success
iopodic acid	success
iopromide	success
iopronic acid	success

iopydol	success
iotalamic acid	success
iotrolan	success
iotroxic	time exceeded
ioversol	success
ioxaglic	time exceeded
ioxilan	success
ioxitalamic acid	success
ipidacrine	success
ipragliflozin	success
ipratropium	success
iprazochrome	success
ipriflavone	success
iprindole	success
iproclozide	success
iproheptine	success
iproniazid	success
irbesartan	success
irinotecan	success
irofulven	success
iron	Antechamber failed
irsogladine	success
isavuconazonium	time exceeded
isaxonine	success
isepamicin	success
isoaminile	success
isobromindione	success
isocarboxazid	success
isoconazole	success
isoetarine	success
isofezolac	success
isoflupredone acetate	success
isoflurane	success

isoleucine	success
isomethadone	success
isometheptene	success
isoniazid	success
isonixin	success
isoprenaline	success
isopromethazine	success
isopropamide	success
isopropanol	success
isosorbide	success
isosorbide dinitrate	success
isosorbide mononitrate	more than one molecule detected
isothipendyl	success
isotretinoin	success
isovaleryl diethylamide	success
isoxepac	success
isoxicam	success
isoxsuprine	success
isradipine	success
istradefylline	success
itopride	success
itraconazole	success
itramin tosilate	more than one molecule detected
ivabradine	success
ivacaftor	success
ivosidenib	success
ixabepilone	success
ixazomib	intramolecular bonds
josamycin	success
kainic acid	success
kanamycin	success

kebuzone	success
keracyanin	more than one molecule detected
ketamine	success
ketanserin	success
ketazolam	success
ketobemidone	success
ketoconazole	success
ketoprofen	success
ketorolac	success
ketotifen	success
ketoxal	success
khellin	success
kinetin	success
kojic acid	success
krypton	intramolecular bonds
labetalol	success
lacidipine	success
lacosamide	success
lactic acid	success
lactitol	success
lactose	success
lactulose	success
lafutidine	success
lamivudine	success
lamotrigine	success
lanatoside	time exceeded
landiolol	success
laninamivir	time exceeded
lanoconazole	success
lanreotide	time exceeded
lansoprazole	success
lapatinib	success

lapyrium	time exceeded
laquinimod	success
laropiprant	success
larotrectinib	success
lascufloxacin	success
lasmiditan	success
lasofoxifene	success
latamoxef	success
latanoprost	success
latanoprostene bunod	more than one molecule detected
lauroguadine	success
ledipasvir	success
lefamulin	success
lefetamine	success
leflunomide	success
leiopyrrole	success
lemborexant	success
lenalidomide	success
lenampicillin	success
lenvatinib	success
lercanidipine	success
lesinurad	success
leteprinim	success
letermovir	success
letosteine	success
letrozole	success
leucine	success
leucinocaine	success
leucovorin	time exceeded
leuprorelin	success
levallorphan	success
levamfetamine	success

levamisole	success
levamlodipine	success
levdobutamine	success
levetiracetam	success
levisoprenaline	success
levmetamfetamine	success
levobetaxolol	success
levobunolol	success
levobupivacaine	success
levocabastine	success
levocarnitine	success
levocarnitine propionate	more than one molecule detected
levocetirizine	success
levodopa	success
levodropropizine	success
levofacetoperane	success
levofloxacin	success
levofolinic acid	time exceeded
levomefolic	time exceeded
levomenol	success
levomenthol	success
levomepromazine	success
levomethadone	success
levomethadyl acetate	success
levomilnacipran	success
levomoprolol	success
levonorgestrel	success
levopropoxyphene	success
levornidazole	success
levorphanol	success
levosalbutamol	success
levosimendan	success

levosulpiride	success
levothyroxine	success
levoverbenone	success
lidamidine	success
lidocaine	success
lidofenin	success
lidoflazine	success
lifitegrast	success
limaprost	success
linaclotide	time exceeded
linagliptin	success
lincomycin	success
lindane	success
linezolid	success
linoleic acid	success
linolenic	time exceeded
linopirdine	success
linsidomine	success
liothyronine	success
liraglutide	intramolecular bonds
liranaftate	success
lisdexamfetamine	success
lisinopril	more than one molecule detected
lisuride	success
lividomycin	success
lixisenatide	time exceeded
lobeglitazone	success
lobenzarit	success
lodoxamide	success
lofepramine	success
lofexidine	success
loflucarban	success

lomefloxacin	success
lomerizine	success
lomifylline	success
lomitapide	success
lomustine	success
lonafarnib	success
lonazolac	success
lonidamine	success
loperamide	success
loperamide oxide	more than one molecule detected
lopinavir	success
loprazolam	success
loprodiol	success
loracarbef	success
lorajmine	success
loratadine	success
lorazepam	success
lorcainide	success
lorcaserin	success
loretin	success
lorlatinib	success
lormetazepam	success
lornoxicam	success
lorpiprazole	success
losartan	success
loteprednol etabonate	success
lovastatin	success
loxapine	success
loxiglumide	success
loxoprofen	success
lubeluzole	success
lubiprostone	success

lucanthone	success
lucimycin	success
luliconazole	success
lumacaftor	success
lumateperone	success
lumefantrine	success
lumiracoxib	success
lurasidone	success
lurbinectedin	success
luseogliflozin	success
lusutrombopag	success
lutein	success
lycopene	success
lymecycline	success
lynestrenol	success
lypressin	intramolecular bonds
lysergide	success
lysine	success
mabuprofen	success
mabuterol	success
macimorelin	success
macitentan	success
mafenide	success
magnesium	intramolecular bonds
magnesium oxide	success
magnesium peroxide	success
malathion	success
malotilate	success
maltose	success
mandelic acid	success
manidipine	success
mannite hexanitrate	more than one molecule detected

mannitol	success
mannomustine	success
mannosulfan	success
maprotiline	success
maraviroc	success
masoprocol	success
maxacalcitol	success
mazaticol	success
mazindol	success
mazipredone	success
mebanazine	success
mebendazole	success
mebeverine	success
mebhydrolin	success
mebicar	success
mebrofenin	success
mebutamate	success
mebutizide	success
mecamylamine	success
mechlorethamine	success
mecillinam	success
meclocycline	success
meclofenamic acid	success
meclofenoxate	success
mecloqualone	success
mecloralurea	success
mecloxamine	success
meclozine	success
mecysteine	success
medazepam	success
medetomidine	success
medibazine	success
medifoxamine	success

medrogestone	success
medroxalol	success
medroxyprogesterone	success
medrylamine	success
medrysone	success
mefenamic acid	success
mefenorex	success
mefexamide	success
mefloquine	success
mefruside	success
mefuparib	success
megestrol acetate	success
meglumine	success
meglutol	success
meladrazine	success
melarsoprol	intramolecular bonds
melatonin	success
meldonium	success
melevodopa	success
melinamide	success
melitracen	success
meloxicam	success
melperone	success
melphalan	success
melphalan flufenamide	success
memantine	success
menadiol sodium diphosphate	success
menadiol sulfate	success
menadione	success
menadione sodium bisulfite	success
menadoxime	success
menatetrenone	success
menbutone	success

menthyl salicylate	success
meobentine	success
mepacrine	success
mepenzolate	success
mephenesin	success
mephenesin carbamate	success
mephenoxalone	success
mephentermine	success
mephenytoin	success
mepindolol	success
mepiperphenidol	success
mepiprazole	success
mepitiostane	success
mepivacaine	success
mepixanox	success
meprednisone	success
meprobamate	success
meproscillarin	success
meprotixol	success
meprylcaine	success
meptazinol	success
mepyramine	success
mequinol	success
mequitazine	success
meradimate	success
mercaptamine	success
mercaptopurine	success
mercuric	intramolecular bonds
mercuric	intramolecular bonds
mercuric	more than one molecule detected
meropenem	success
mesalazine	success

mesna	success
mesoridazine	success
mestanolone	success
mesterolone	success
mestranol	success
mesulfen	success
metabutethamine	success
metabutoxycaine	success
metaclazepam	success
metacycline	success
metahexamide	success
metamfepramone	success
metamizole	success
metampicillin	success
metandienone	success
metapramine	success
metaraminol	success
metaxalone	success
metergoline	success
metesculetol	success
metformin	success
methacholine	success
methadone	success
methallatal	success
methallenestril	success
methamphetamine	success
methandriol	success
methaniazide	success
methanthelinium	success
methaphenilene	success
methapyrilene	success
methaqualone	success
metharbital	success

methazolamide	success
methdilazine	success
methenamine	success
methenolone	success
methestrol	success
methestrol dipropionate	success
methiodal	success
methionine	success
methiosulfonium chloride	more than one molecule detected
methitural	success
methocarbamol	success
methohexital	success
methopromazine	success
methoserpidine	success
methotrexate	more than one molecule detected
methoxamine	success
methoxsalen	success
methoxyflurane	success
methoxyphenamine	success
methscopolamine	success
methsuximide	success
methyl aminolevulinate	success
methyl nicotinate	success
methyl salicylate	success
methylatropine	success
methylchromone	success
methylclothiazide	success
methyldopa	success
methyldopate	success
methylephedrine	success
methylergometrine	success

methylestrenolone	success
methylhexaneamine	success
methylhomatropine	success
methylnaltrexone	success
methylparaben	success
methylpentynol	success
methylphenidate	success
methylphenobarbital	success
methylprednisolone	success
methylprednisolone	time exceeded
methylprednisolone	time exceeded
methylprednisolone	time exceeded
methylprednisolone acetate	success
methylrosaniline	success
methylsulfonal	success
methyltestosterone	success
methylthioninium chloride	more than one molecule detected
methylthiouracil	success
methyprylon	success
methysergide	success
metiazinic acid	success
meticillin	success
meticrane	success
metildigoxin	time exceeded
metipamide	success
metipranolol	success
metirosine	success
metisazone	success
metixene	success
metizoline	success
metochalcone	success
metoclopramide	success

metofenazate	success
metofoline	success
metolazone	success
metopimazine	success
metopon	success
metoprolol	success
metralindole	success
metrifonate	success
metrizamide	success
metrizoic	intramolecular bonds
metronidazole	success
meturedepa	success
metyrapone	success
mexazolam	success
mexenone	success
mexiletine	success
mezlocillin	success
mianserin	success
mibefradil	success
micafungin	intramolecular bonds
micinicate	success
miconazole	success
micronomicin	success
midazolam	success
midodrine	success
midostaurin	success
mifamurtide	time exceeded
mifepristone	success
migalastat	success
miglitol	success
miglustat	success
milnacipran	success
miloxacin	success

milrinone	success
miltefosine	success
milverine	success
mimosine	success
minaprine	success
minocycline	success
minodronic acid	success
minoxidil	success
miocamycin	time exceeded
mirabegron	success
mirodenafil	success
mirtazapine	success
misoprostol	success
mitiglinide	success
mitobronitol	success
mitoguazone	success
mitolactol	success
mitomycin	success
mitopodozide	success
mitotane	success
mitoxantrone	success
mivacurium	success
mivotilate	success
mizolastine	success
mizoribine	success
moclobemide	success
modafinil	success
moexipril	more than one molecule detected
mofebutazone	success
mofezolac	success
molindone	success
molsidomine	success

Molybdenum	time exceeded
mometasone furoate	success
monobenzone	success
monoctanoin	success
monoxerutin	success
montelukast	success
moperone	success
mopidamol	success
moprolol	success
moquizone	success
moracizine	success
morazone	success
morclofone	success
morinamide	success
morinidazole	success
morniflumate	success
moroxydine	success
morphine	success
mosapramine	success
mosapride	success
motretinide	success
moxastine	success
moxaverine	success
moxestrol	success
moxidectin	intramolecular bonds
moxifloxacin	success
moxisylyte	success
moxonidine	success
mozavaptan	success
mupirocin	success
muzolimine	success
mycophenolate mofetil	success
mycophenolic acid	success

mydecamycin	success
myrophine	success
myrtecaine	success
N-Acetylglucosamine	success
N-Acetyltyrosine	more than one molecule detected
nabilone	success
nabumetone	success
nadide	more than one molecule detected
nadifloxacin	success
nadolol	success
nadoxolol	success
naepaine	success
nafamostat	success
nafarelin	success
nafcillin	success
nafiverine	success
naftazone	success
naftidrofuryl	success
naftifine	success
naftopidil	success
nalbuphine	success
naldemedine	success
nalfurafine	success
nalidixic acid	success
nalmefene	success
nalorphine	success
naloxegol	success
naloxone	success
naltrexone	success
nandrolone	success
nandrolone	time exceeded

nandrolone	time exceeded
nandrolone cyclotate	success
naphazoline	success
naproxcinod	success
naproxen	success
naratriptan	success
narcobarbital	success
natamycin	success
nateglinide	more than one molecule detected
nealbarbital	success
nebivolol	success
nedocromil	success
nefazodone	success
nefiracetam	success
nefopam	success
nelarabine	success
nelfinavir	success
neltenexine	success
nemonapride	success
nemonoxacin	success
neostigmine	success
nepafenac	success
nepinalone	success
neratinib	success
neridronic acid	success
nesiritide	time exceeded
netarsudil	success
neticonazole	success
netilmicin	success
netupitant	success
nevirapine	success
nialamide	success

niaprazine	success
nicametate	success
nicardipine	success
nicergoline	success
niceritrol	success
niclofolan	success
niclosamide	success
nicoclonate	success
nicocodine	success
nicofetamide	success
nicofibrate	success
nicofuranose	success
nicomol	success
nicomorphine	success
nicorandil	success
nicotinamide	success
nicotine	success
nicotinic acid	success
nicotinyl alcohol	success
nicotinyl methylamide	success
nidroxyzone	success
nifedipine	success
nifekalant	success
nifenalol	success
nifenazone	success
niflumic acid	success
nifuradene	success
nifuratel	success
nifurfoline	success
nifuroxazide	success
nifuroxime	success
nifurpirinol	success
nifurprazine	success

nifurtimox	success
nifurtoinol	success
nifurzide	success
nikethamide	success
nilotinib	success
nilutamide	success
nilvadipine	success
nimesulide	success
nimetazepam	success
nimodipine	success
nimorazole	success
nimustine	success
nintedanib	success
niperotidine	success
nipradilol	success
niraparib	success
niridazole	success
nisoldipine	success
nitazoxanide	success
nitisinone	success
nitracrine	success
nitrazepam	success
nitrefazole	success
nitrendipine	success
nitric	Antechamber failed
nitrofurantoin	success
nitrofurazone	success
nitrogen	success
nitrous oxide	more than one molecule detected
nitroxazepine	success
nitroxoline	success
nizatidine	success

nizofenone	success
nomegestrol	success
nomegestrol acetate	success
nomifensine	success
nordazepam	success
nordefrin	success
norelgestromin	success
norepinephrine	success
norethandrolone	success
norethindrone	time exceeded
norethindrone acetate	success
norethisterone	success
norethynodrel	success
norfenefrine	success
norfloxacin	success
norgesterone	success
norgestimate	success
norgestrienone	success
normethadone	success
norpipanone	success
nortriptyline	success
norvinisterone	success
noscapine	success
novobiocin	success
noxiptiline	success
noxytiolin	success
nystatin	success
obeticholic acid	success
obidoxime	success
octabenzone	success
octacaine	success
octamoxin	success
octamylamine	success

octanoic acid	success
octatropine methylbromide	more than one molecule detected
octenidine	success
octinoxate	success
octisalate	success
octocrylene	success
octodrine	success
octopamine	success
octotiamine	success
octreotide	time exceeded
octyltriethoxysilane	success
ofloxacin	success
oftasceine	success
olaflur	success
olanexidine	time exceeded
olanzapine	success
olaparib	success
oleandomycin	success
oleandrin	success
oleic acid	success
oliceridine	success
olmesartan	time exceeded
olmutinib	success
olodaterol	success
olopatadine	success
olprinone	success
olsalazine	success
omacetaxine mepesuccinate	success
omadacycline	success
omapatrilat	success
omarigliptin	success
ombitasvir	success

omeprazole	success
omidenepag	time exceeded
omoconazole	success
ondansetron	success
opicapone	success
opipramol	success
orazamide	success
orciprenaline	success
oritavancin	more than one molecule detected
orlistat	success
ormeloxifene	success
ornidazole	success
ornipressin	intramolecular bonds
ornithine	success
ornoprostil	success
orotic acid	success
orphenadrine	success
orthocaine	success
osalmid	success
oseltamivir	success
osilodrostat	success
osimertinib	success
ospemifene	success
oteracil	success
otilonium bromide	more than one molecule detected
ouabain	success
oxabolone	success
oxabolone cipionate	success
oxaceprol	success
oxacillin	success
oxaflozane	success

oxaflumazine	success
oxagrelate	success
oxamarin	success
oxametacin	success
oxamniquine	success
oxanamide	success
oxandrolone	success
oxantel	success
oxapropanium	success
oxaprozin	success
oxatomide	success
oxazepam	success
oxazolam	success
oxcarbazepine	success
oxedrine	success
oxeladin	success
oxendolone	success
oxetacaine	success
oxetorone	success
oxiconazole	success
oxidronic acid	success
oxiglutatione	more than one molecule detected
oxilofrine	success
oxiniacic acid	more than one molecule detected
oxiracetam	success
oxitriptan	success
oxitropium	success
oxolamine	success
oxolinic acid	success
oxomemazine	success
oxophenarsine	Antechamber failed

oxprenolol	success
oxybenzone	success
oxybuprocaine	success
oxybutynin	success
oxycinchophen	success
oxyclipine	success
oxycodone	success
oxydibutanol	success
oxyfedrine	success
oxyfenamate	success
oxygen	success
oxymesterone	success
oxymetazoline	success
oxymetholone	success
oxymethurea	success
oxymorphone	success
oxypendyl	success
oxypertine	success
oxyphenbutazone	success
oxyphencyclimine	success
oxyphenisatine	success
oxyphenisatine acetate	success
oxyphenonium	success
oxyquinoline	success
oxysonium	more than one molecule detected
oxytetracycline	success
oxytocin	time exceeded
ozagrel	success
ozanimod	success
ozenoxacin	success
paclitaxel	success
Padimate A	success

Padimate O	success
palbociclib	success
paliperidone	success
paliperidone	time exceeded
palmidrol	success
palonosetron	success
pamabrom	success
pamaquine	success
pamidronic acid	success
pancuronium	success
panipenem	success
panobinostat	success
pantethine	success
pantoprazole	success
papaverine	success
papaveroline	success
paracetamol	success
paraldehyde	success
paramethadione	success
paramethasone acetate	success
paraoxon	success
parapenzolate	success
parathiazine	success
parecoxib	success
parethoxycaine	success
pargeverine	success
pargyline	success
paricalcitol	success
paritaprevir	success
paromomycin	success
paroxetine	success
paroxypropione	success
parsalmide	success

pasireotide	more than one molecule detected
pazopanib	success
pazufloxacin	success
pecazine	success
pecilocin	success
pefloxacin	success
pemafibrate	success
pemetrexed	more than one molecule detected
pemigatinib	success
pemirolast	success
pemoline	success
pempidine	success
penamecillin	success
penbutolol	success
penciclovir	success
penfluridol	success
penicillamine	success
penmesterol	success
pentacynium chloride	more than one molecule detected
pentaerithrityl tetranitrate	more than one molecule detected
pentaerythritol	success
pentagastrin	success
pentagestrone	success
pentagestrone acetate	success
pentamethonium bromide	more than one molecule detected
pentamidine	success
pentamycin	time exceeded
pentapiperium metilsulfate	more than one molecule detected
pentazocine	success

pentetic acid	success
pentetrazol	success
penthienate	success
pentifylline	success
pentobarbital	success
pentolonium	success
pentorex	success
pentostatin	success
pentoxifylline	success
pentoxyverine	success
pentrinitrol	success
peplomycin	success
peramivir	success
perampanel	success
perazine	success
perflenapent	success
perflexane	success
perfluamine	success
perflubron	success
perflubutane	success
perflunafene	success
perflutren	success
perfosfamide	success
pergolide	success
perhexiline	success
periciazine	success
perimetazine	success
perindopril	more than one molecule detected
perisoxal	success
perlapine	success
permethrin	success
perospirone	success

perphenazine	success
peruvoside	success
pethidine	success
petrichloral	success
pexidartinib	success
phanquinone	success
phenacaine	success
phenacemide	success
phenacetin	success
phenadoxone	success
phenaglycodol	success
phenallymal	success
phenamacide	success
phenamine	success
phenazocine	success
phenazone	success
phenazopyridine	success
phenbenzamine	success
phenbutamide	success
phencyclidine	success
phendimetrazine	success
phenelzine	success
pheneticillin	success
pheneturide	success
phenformin	success
phenglutarimide	success
phenibut	success
phenicarbazide	success
phenindamine	success
phenindione	success
pheniodol sodium	success
pheniprazine	success
pheniramine	success

phenmetrazine	success
phenobarbital	success
phenobutiodil	success
phenol	success
phenolphthalein	success
phenolphthalol	success
phenolsulfonphthalein	success
phenoperidine	success
phenosulfazole	success
phenothrin	success
phenoxybenzamine	success
phenoxyethanol	success
phenoxymethylpenicillin	success
phenprobamate	success
phenprocoumon	success
phenpromethamine	success
phensuximide	success
phentermine	success
phentetramine	success
phentolamine	success
phenyl acetylsalicylate	success
phenyl salicylate	success
phenylacetic acid	success
phenylalanine	success
phenylbutanoic acid	success
phenylbutazone	success
phenylephrine	success
phenylethanolamine	success
phenylmercuric nitrate	more than one molecule detected
phenylpropanol	success
phenylpropanolamine	success
phenyltoloxamine	success

phenytoin	success
phetharbital	success
phethenylate	success
phloroglucinol	success
pholcodine	success
pholedrine	success
phosphocreatine	success
phosphoric acid	success
phosphorylcholine	success
phthalylsulfacetamide	success
phthalylsulfathiazole	success
physostigmine	success
phytomenadione	success
piberaline	success
pibrentasvir	success
picilorex	success
picloxydine	success
picodralazine	success
picoperine	success
picotamide	success
picrotin	success
pidotimod	success
pifarnine	success
piketoprofen	success
pildralazine	success
pilocarpine	success
pilsicainide	success
pimavanserin	success
pimeclone	success
pimecrolimus	success
pimefylline	success
pimethixene	success
piminodine	success

pimobendan	success
pimozide	success
pinacidil	success
pinaverium	success
pinazepam	success
pindolol	success
pioglitazone	success
pipamazine	success
pipamperone	success
pipazetate	success
pipebuzone	success
pipecuronium	success
pipemidic acid	success
pipenzolate	success
piperacetazine	success
piperacillin	success
piperaquine	success
piperazine	success
piperidione	success
piperidolate	success
piperonyl butoxide	success
pipethanate	success
pipobroman	success
piposulfan	success
pipotiazine	time exceeded
pipoxolan	success
pipradrol	success
piprozolin	success
piracetam	success
pirarubicin	success
pirbuterol	success
pirenoxine	success
pirenzepine	success

piretanide	success
pirfenidone	success
piribedil	success
piridocaine	success
pirifibrate	success
pirisudanol	success
piritramide	success
pirlindole	success
pirmenol	success
piroctone	success
piroheptine	success
piromidic acid	success
piroxicam	success
pirozadil	success
pirprofen	success
pitavastatin	success
pitofenone	success
pitolisant	success
pivagabine	success
pivampicillin	success
pivhydrazine	success
pivmecillinam	success
pixantrone	success
pizotifen	success
plafibride	success
plaunotol	success
plazomicin	success
plecanatide	more than one molecule detected
pleconaril	success
plerixafor	success
plicamycin	success
podophyllotoxin	success

poldine	success
polmacoxib	success
polydatin	success
polythiazide	success
pomalidomide	success
ponatinib	success
ponesimod	success
posaconazole	success
posatirelin	success
potassium canrenoate	success
practolol	success
prajmalium	success
pralatrexate	time exceeded
pralidoxime	more than one molecule detected
pralmorelin	success
pralsetinib	success
pramipexole	success
pramiracetam	success
pramiverine	success
pramlintide	time exceeded
pramocaine	success
pranlukast	success
pranoprofen	success
prasterone	success
prasterone acetate	success
prasterone sulfate	success
prasugrel	success
pravastatin	success
prazepam	success
praziquantel	success
prazosin	success
prednicarbate	success

prednimustine	success
prednisolamate	success
prednisolone	success
prednisolone	time exceeded
prednisolone	time exceeded
prednisolone	time exceeded
prednisolone	time exceeded
prednisolone	time exceeded
prednisolone acetate	success
prednisolone sodium phosphate	success
prednisolone sulfobenzoate	success
prednisone	success
prednival	success
prednylidene	success
pregabalin	success
pregnenolone succinate	success
prenalterol	success
prenderol	success
prenoxdiazine	success
prenylamine	success
pretomanid	success
pridinol	success
prifinium	success
prilocaine	success
primaquine	success
primidone	success
probenecid	success
probucol	success
procainamide	success
procaine	success
procarbazine	success
procaterol	success
prochlorperazine	success

procodazole	success
procyclidine	success
procymate	success
profenamine	success
proflavine	success
progabide	success
progesterone	success
proglumetacin	success
proglumide	success
proguanil	success
proline	success
prolintane	success
promazine	success
promegestone	success
promestriene	success
promethazine	success
promoxolane	success
pronetalol	success
pronilide	success
propacetamol	success
propafenone	success
propallylonal	success
propamidine	success
propanidid	success
propanol	success
propantheline	success
proparacaine	success
propatylnitrate	success
propenidazole	success
propentofylline	success
propicillin	success
propiolactone	success
propiomazine	success

propipocaine	success
propiram	success
propiverine	success
propizepine	success
propofol	success
propoxycaine	success
propranolol	success
propylene glycol	success
propylhexedrine	success
propyliodone	success
propylparaben	success
propylthiouracil	success
propyphenazone	success
propyromazine	success
proquazone	success
proscillaridin	success
protheobromine	success
prothipendyl	success
protiofate	success
protionamide	success
protirelin	success
protizinic acid	success
protocatechualdehyde	success
protokylol	success
protoporphyrin	success
protoverine	success
protriptyline	success
proxazole	success
proxibarbal	success
proxyphylline	success
prozapine	success
prucalopride	success
prulifloxacin	success

pseudoephedrine	success
pyrantel	success
pyrazinamide	success
pyricarbate	success
pyridostigmine	more than one molecule detected
pyridoxal	success
pyridoxal phosphate	success
pyridoxine	success
pyrimethamine	success
pyrinoline	success
pyrithione	success
pyrithyldione	success
pyritinol	success
pyronaridine	success
pyrovalerone	success
pyrrobutamine	success
pyrrocaine	success
pyrrolnitrin	success
pyrvinium	more than one molecule detected
quazepam	success
quercetin	success
quetiapine	success
quifenadine	success
quinagolide	success
quinapril	more than one molecule detected
quinbolone	success
quinestradol	success
quinestrol	success
quinethazone	success
quinfamide	success
quingestanol acetate	success

quinidine	success
quinine	success
quinisocaine	success
quinupramine	success
quinupristin	success
quizartinib	success
rabeprazole	success
racecadotril	success
racepinefrine	success
radium	Antechamber failed
radotinib	success
raloxifene	success
raltegravir	success
raltitrexed	time exceeded
ramatroban	success
ramelteon	success
ramifenazone	success
ramipril	more than one molecule detected
ramosetron	success
ranimustine	success
ranitidine	success
ranolazine	success
rapacuronium	success
rasagiline	success
razoxane	success
rebamipide	success
reboxetine	success
regadenoson	success
regorafenib	success
relebactam	success
relugolix	success
remdesivir	success

remifentanil	success
remikiren	success
remimazolam	success
remoxipride	success
repaglinide	success
repirinast	success
reposal	success
reproterol	success
rescimetol	success
rescinnamine	success
reserpiline	success
reserpine	success
resorcinol	success
retapamulin	success
retigabine	success
retinol	success
retinol acetate	success
revaprazan	success
revefenacin	success
rhodoquine	success
ribavirin	success
ribociclib	success
riboflavin	success
riboflavin phosphate	success
ribostamycin	success
ricinoleic acid	success
ridaforolimus	intramolecular bonds
rifabutin	intramolecular bonds
rifampicin	success
rifamycin	success
rifapentine	success
rifaximin	time exceeded
rilmazafone	success

rilmenidine	success
rilpivirine	success
riluzole	success
rimantadine	success
rimazolium	more than one molecule detected
rimegepant	success
rimexolone	success
rimiterol	success
rimonabant	success
riociguat	success
riodoxol	success
rioprostil	success
ripasudil	success
ripazepam	success
ripretinib	success
risdiplam	success
risedronic acid	success
risperidone	success
ritiometan	success
ritodrine	success
ritonavir	success
rivaroxaban	success
rivastigmine	success
rizatriptan	success
rociverine	success
rocuronium	success
rofecoxib	success
roflumilast	success
rokitamycin	success
rolapitant	success
rolitetracycline	success
romidepsin	success

romurtide	success
ronifibrate	success
ropinirole	success
ropivacaine	success
roquinimex	success
rosaprostol	success
rosiglitazone	success
rosoxacin	success
rosuvastatin	success
rotigotine	success
rotraxate	success
roxadustat	more than one molecule detected
roxatidine acetate	success
roxithromycin	time exceeded
rubitecan	success
ruboxistaurin	success
rucaparib	success
rufinamide	success
rufloxacin	success
rupatadine	success
rutoside	success
ruxolitinib	success
sabcomeline	success
sacubitril	success
safinamide	success
salacetamide	success
salamidacetic acid	success
salazosulfadimidine	success
salbutamol	success
salicylamide	success
salicylanilide	success
salicylic acid	success

salicylsulfuric acid	success
salinazid	success
salmeterol	success
salsalate	success
samidorphan	success
sancycline	success
sapropterin	success
saquinavir	success
saralasin	more than one molecule detected
sarecycline	success
saroglitazar	success
sarpogrelate	success
saxagliptin	success
scarlet red	success
scopolamine	success
scopolamine butylbromide	more than one molecule detected
secalciferol	success
secbutabarbital	success
secnidazole	success
secobarbital	success
secretin	time exceeded
secretin porcine	more than one molecule detected
selegiline	success
selenium (75Se) tauroselcholic acid	success
selenium sulfide	success
selenomethionine	success
selexipag	success
selinexor	success
selpercatinib	success
selumetinib	success

semaglutide	more than one molecule detected
semustine	success
sequifenadine	success
seractide	more than one molecule detected
seratrodast	success
serdexmethylphenidate	more than one molecule detected
serine	success
sermorelin	time exceeded
sertaconazole	success
sertindole	success
sertraline	success
setastine	success
setiptiline	success
setmelanotide	intramolecular bonds
sevoflurane	success
sibutramine	success
siccanin	success
sildenafil	success
silicon dioxide	success
silodosin	success
simeprevir	success
simetride	success
simfibrate	success
simvastatin	success
sincalide	success
siponimod	success
sirolimus	intramolecular bonds
sisomicin	success
sitafloxacin	success
sitagliptin	success
sitaxentan	success

sitosterol	success
sivelestat	success
sobrerol	success
sobuzoxane	success
sodium gualenate	success
sodium hexacyclonate	success
sodium myristyl sulfate	success
sodium stibogluconate	success
sodium tetradecyl sulfate	success
sodium thiosalicylate	success
sofalcone	success
sofosbuvir	success
sofpironium bromide	more than one molecule detected
solasulfone	success
solifenacin	success
solriamfetol	success
somatostatin	time exceeded
sonidegib	success
sorafenib	success
sorbinicate	success
sorbitol	success
sorivudine	success
sotagliflozin	success
sotalol	success
sotorasib	more than one molecule detected
sozoiodolic acid	success
spaglumic acid	success
sparfloxacin	success
sparteine	success
spectinomycin	success
spiclomazine	success

spiperone	success
Spiramycin	time exceeded
Spiramycin	time exceeded
Spiramycin	time exceeded
spirapril	time exceeded
spironolactone	success
spizofurone	success
stallimycin	success
stannous fluoride	success
stanozolol	success
stavudine	success
stearic acid	success
stepronin	success
stiripentol	success
streptomycin	success
Streptomycin	time exceeded
streptozocin	success
strychnine	success
styramate	success
subathizone	success
succimer	success
succinic acid	success
succinimide	success
succinylsulfathiazole	success
succisulfone	success
suclofenide	success
sucrose	success
sufentanil	success
sugammadex	success
sulbactam	success
sulbactam pivoxyl	success
sulbenicillin	success
sulbentine	success

sulbutiamine	success
sulcaine	success
sulconazole	success
sulfabenzamide	success
sulfacarbamide	success
sulfacetamide	success
sulfachlorpyridazine	success
sulfachrysoidine	success
sulfacytine	success
sulfadiasulfone	success
sulfadiazine	success
sulfadicramide	success
sulfadimethoxine	success
sulfadimidine	success
sulfadoxine	success
sulfaethidole	success
sulfafurazole	success
sulfaguanidine	success
sulfaguanole	success
sulfalene	success
sulfaloxic acid	success
sulfamazone	success
sulfamerazine	success
sulfameter	success
sulfamethizole	success
sulfamethoxazole	success
sulfamethoxypyridazine	success
sulfamethylthiazole	success
sulfametomidine	success
sulfametrole	success
sulfamidochrysoidine	success
sulfamonomethoxine	success
sulfamoxole	success

sulfanilamide	success
sulfaperin	success
sulfaphenazole	success
sulfapyridine	success
sulfarside	Antechamber failed
sulfasalazine	success
sulfasomizole	success
sulfasymazine	success
sulfathiazole	success
sulfathiourea	success
sulfinalol	success
sulfinpyrazone	success
sulfiram	success
sulfisomidine	success
sulfisoxazole acetyl	success
sulfobromophthalein	success
sulforidazine	success
sulfur	success
sulfur hexafluoride	success
sulindac	success
sulisatin	success
sulisobenzone	success
sulmarin	success
sulmazole	success
suloctidil	success
sulpiride	success
sulprostone	success
sultamicillin	success
sultiame	success
sultopride	success
sultosilic acid	success
sumatriptan	success
sunitinib	success

suplatast	more than one molecule detected
suprofen	success
suramin	success
suvorexant	success
suxamethonium	success
suxibuzone	success
symclosene	success
syrosingopine	success
tacalcitol	success
tacrine	success
tacrolimus	success
tadalafil	success
tafamidis	success
tafenoquine	success
tafluprost	success
talampicillin	success
talaporfin	time exceeded
talastine	success
talazoparib tosylate	success
talbutal	success
talinolol	success
talipexole	success
talniflumate	success
taltirelin	success
tamibarotene	success
tamoxifen	success
tamsulosin	success
tandospirone	success
tapentadol	success
taribavirin	success
tartaric acid	success
tartrazine	success

tasimelteon	success
tasosartan	success
taurine	success
taurolidine	success
taurosteine	success
tavaborole	Antechamber failed
tazanolast	success
tazarotene	success
tazemetostat	success
tazobactam	success
tebipenem pivoxil	success
teclothiazide	success
teclozan	success
tecovirimat	success
tedisamil	success
tedizolid phosphate	success
teduglutide	more than one molecule detected
teflurane	success
tegafur	success
tegaserod	success
teicoplanin aglycone	more than one molecule detected
telaprevir	success
telavancin	time exceeded
telbivudine	success
telithromycin	success
telmesteine	success
telmisartan	success
telotristat	time exceeded
temafloxacin	success
temazepam	success
temocapril	success

temocillin	success
temoporfin	success
temozolomide	success
temsirolimus	intramolecular bonds
tenapanor	time exceeded
teneligliptin	success
tenidap	success
teniposide	success
tenitramine	success
tenofovir	success
tenofovir	time exceeded
tenofovir	time exceeded
tenonitrozole	success
tenoxicam	success
tepotinib	success
teprenone	success
terazosin	success
terbinafine	success
terbutaline	success
terconazole	success
terfenadine	success
terguride	success
teriflunomide	success
teriparatide	more than one molecule detected
terizidone	success
terlipressin	intramolecular bonds
terodiline	success
terpin	success
tertatolol	success
testolactone	success
testosterone	success
testosterone	time exceeded

testosterone	time exceeded
testosterone cypionate	success
testosterone propionate	success
tetrabarbital	success
tetrabenazine	success
tetracaine	success
tetrachloroethylene	success
tetracosactide	more than one molecule detected
tetracycline	success
tetraethylphthalamide	success
tetragalacturonic	time exceeded
tetramethrin	success
tetrazepam	success
tetrofosmin	success
tetroxoprim	success
tetryzoline	success
tevenel	success
tezacaftor	success
thalidomide	success
thallous	Antechamber failed
thebacon	success
thenalidine	success
theobromine	success
theodrenaline	success
theophylline	success
thiabendazole	success
thialbarbital	success
thiamazole	success
thiamine	more than one molecule detected
thiamphenicol	success
thiamphenicol glycinate	success

thiamylal	success
thiazinam	success
thiazosulfone	success
thiethylperazine	success
thioacetazone	success
thiocolchicoside	success
thioctic acid	success
thioguanine	success
thiopental	success
thiopropazate	success
thioproperazine	success
thioridazine	success
thiotepa	success
thiothixene	success
thiouracil	success
thiram	success
thonzylamine	success
threonine	success
thurfyl nicotinate	success
thymalfasin	time exceeded
thymol	success
thymopentin	more than one molecule detected
thyropropic acid	success
tiadenol	success
tiagabine	success
tiamenidine	success
tiamiprine	success
tianeptine	success
tiapride	success
tiaprofenic acid	success
tiaramide	success
tiazesim	success

tiazofurine	success
tibezonium	success
tibolone	success
ticagrelor	success
ticarcillin	success
ticlatone	success
ticlopidine	success
tidiacic	success
tiemonium	success
tienilic acid	success
tifenamil	success
tigecycline	success
tigemonam	success
tigloidine	success
tilbroquinol	success
tilidine	success
tiliquinol	success
tilisolol	success
tiludronic acid	success
timepidium	success
timiperone	success
timolol	success
timonacic	success
tinazoline	success
tinidazole	success
tinofedrine	success
tinoridine	success
tiocarlide	success
tioclomarol	success
tioconazole	success
tiomesterone	success
tiopronin	success

tiotropium bromide	more than one molecule detected
tioxolone	success
tipepidine	success
tipiracil	success
tipranavir	success
tiquizium	success
tirabrutinib	time exceeded
tiracizine	success
tiratricol	success
tirbanibulin	success
tirilazad	success
tirofiban	success
tiropramide	success
tisopurine	success
titanium	Antechamber failed
tivozanib	success
tixocortol	success
tizanidine	success
tobramycin	success
tobuterol	success
tocainide	success
tocofersolan	success
tocofibrate	success
tocopherol	time exceeded
tocopheryl	time exceeded
todralazine	success
tofacitinib	success
tofenacin	success
tofisopam	success
tofogliflozin	success
tolazamide	success
tolazoline	success

tolbutamide	success
tolcapone	success
tolciclate	success
tolfenamic acid	success
tolindate	success
toliprolol	success
tolmetin	success
tolnaftate	success
tolonidine	success
tolonium	more than one molecule detected
toloxatone	success
tolperisone	success
tolpropamine	success
tolrestat	success
tolterodine	success
tolvaptan	success
tolycaine	success
tonzonium	success
topiramate	success
topiroxostat	success
topotecan	success
toremifene	success
torsemide	success
tosufloxacin	success
tosylchloramide sodium	success
trabectedin	success
tramadol	success
tramazoline	success
trametinib	success
trandolapril	more than one molecule detected
tranexamic acid	success

tranilast	success
tranylcypromine	success
trapidil	success
travoprost	success
traxanox	success
trazodone	success
trelagliptin	success
trenbolone hexahydrobenzylcarbonate	success
trengestone	success
treosulfan	success
trepibutone	success
treprostinil	success
tretamine	success
tretinoin	success
tretinoin	time exceeded
tretoquinol	success
triacetin	success
triamcinolone	success
triamcinolone	time exceeded
triamcinolone	time exceeded
triamcinolone	time exceeded
triamcinolone acetonide	success
triamterene	success
triaziquone	success
triazolam	success
tribenoside	success
tribromoethanol	success
tribromometacresol	success
tribuzone	success
trichlormethiazide	success
trichloroacetic acid	success
trichloroethylene	success
triclabendazole	success

triclocarban	success
triclofos	success
triclosan	success
tricyclamol	time exceeded
tridihexethyl	success
trientine	success
trifarotene	success
trifluoperazine	success
trifluperidol	success
triflupromazine	success
trifluridine	success
triflusal	success
triheptanoin	success
trihexyphenidyl	success
trilaciclib	success
trilostane	success
trimazosin	success
trimebutine	success
trimecaine	success
trimegestone	success
trimetaphan	more than one molecule detected
trimetazidine	success
trimethadione	success
trimethidinium	success
trimethobenzamide	success
trimethoprim	success
trimethyldiphenylpropylamine	success
trimetozine	success
trimetrexate	success
trimipramine	success
trinitrophenol	success
trioxsalen	success

tripamide	success
triparanol	success
tripelennamine	success
triprolidine	success
triptorelin	success
tritiozine	success
tritoqualine	success
trofosfamide	success
troglitazone	success
trolamine	success
troleandomycin	success
trolnitrate	success
tromantadine	success
trometamol	success
tropatepine	success
tropesin	success
tropicamide	success
tropisetron	success
trospectomycin	success
trospium	success
trovafloxacin	success
troxacitabine	success
troxerutin	success
troxipide	success
trypan	time exceeded
tryparsamide	Antechamber failed
tryptophan	success
tuaminoheptane	success
tubocurarine	success
tucatinib	success
tulobuterol	success
tybamate	success
tymazoline	success

tyramine	success
tyropanoate	intramolecular bonds
tyrosine	success
ubenimex	more than one molecule detected
ubidecarenone	success
ubrogepant	success
udenafil	success
ufenamate	success
ulipristal	success
ulobetasol propionate	success
umbralisib	success
umeclidinium	success
umifenovir	success
undecylenic acid	success
unithiol	success
unoprostone	time exceeded
upadacitinib	success
uracil mustard	success
urapidil	success
UREA C 14	success
UREA C-13	success
uredepa	success
uridine triacetate	success
uridine triphosphate	success
ursodiol	success
vaborbactam	Antechamber failed
vadadustat	time exceeded
valaciclovir	success
valbenazine	success
valdecoxib	success
valdetamide	success
valethamate	success

valganciclovir	success
valine	success
valnoctamide	success
valproic acid	success
valpromide	success
valrubicin	success
valsartan	success
vancomycin	time exceeded
vandetanib	success
vaniprevir	success
vanitiolide	success
vardenafil	success
varenicline	success
vasopressin	intramolecular bonds
vecuronium	success
vedaprofen	success
velnacrine	success
velpatasvir	success
vemurafenib	success
venetoclax	success
venlafaxine	success
veralipride	success
verapamil	success
vericiguat	success
vernakalant	success
vesnarinone	success
vibegron	success
vidarabine	success
vigabatrin	success
vilanterol	success
vilazodone	success
vildagliptin	success
viloxazine	success

viminol	success
vinbarbital	success
vinblastine	success
vinburnine	success
vincamine	success
vincristine	time exceeded
vindesine	success
vinflunine	success
vinorelbine	success
vinpocetine	success
vintafolide	time exceeded
vinyl ether	success
vinylbital	success
viomycin	more than one molecule detected
viquidil	success
vismodegib	success
visnadine	success
vitamin	time exceeded
voacamine	success
voclosporin	intramolecular bonds
voglibose	more than one molecule detected
vonoprazan	success
vorapaxar	success
voriconazole	success
vorinostat	success
vorozole	success
vortioxetine	success
voxelotor	success
voxilaprevir	success
warfarin	success
xamoterol	success

xanomeline	success
xantinol	success
xenbucin	success
xenon	Antechamber failed
xenon (127Xe) gas	success
xenon (133Xe) gas	success
xenysalate	success
xenytropium	success
xibenolol	success
xibornol	success
ximelagatran	success
xipamide	success
xylitol	success
xylometazoline	success
xylose	success
xyloylsulfamine	success
yohimbine	success
zabofloxacin	success
zafirlukast	success
zalcitabine	success
zaleplon	success
zaltoprofen	success
zanamivir	success
zanubrutinib	success
zeranol	success
ziconotide	time exceeded
zidovudine	success
zileuton	success
zimeldine	success
zinc	Antechamber failed
zinostatin	success
zipeprol	success
ziprasidone	success

zofenopril	success
zoledronic acid	success
zolimidine	success
zolmitriptan	success
zolpidem	success
zomepirac	success
zonisamide	success
zopiclone	success
zorubicin	success
zotarolimus	intramolecular bonds
zotepine	success
zoxazolamine	success
zucapsaicin	success
zuclopenthixol	success
zuclopenthixol acetate	success
